# *Arabidopsis* RAD51, RAD51C and XRCC3 proteins form a complex and facilitate RAD51 localization on chromosomes for meiotic recombination

**DOI:** 10.1371/journal.pgen.1006827

**Published:** 2017-05-31

**Authors:** Hang Su, Zhihao Cheng, Jiyue Huang, Juan Lin, Gregory P. Copenhaver, Hong Ma, Yingxiang Wang

**Affiliations:** 1 State Key Laboratory of Genetic Engineering and Collaborative Innovation Center of Genetics and Development, Ministry of Education Key Laboratory of Biodiversity Sciences and Ecological Engineering, Institute of Plant Biology, School of Life Sciences, Fudan University, Shanghai, China; 2 Institute of Banana and Plantain, Chinese Academy of Tropic Agriculture Science, Haikou, Hainan province, China; 3 Department of Biology and the Integrative Program for Biological and Genome Sciences, University of North Carolina at Chapel Hill, Chapel Hill, North Carolina, United States of America; 4 Lineberger Comprehensive Cancer Center, University of North Carolina School of Medicine, Chapel Hill, North Carolina, United States of America; 5 Center for Evolutionary Biology, Institutes of Biomedical Sciences, School of Life Sciences, Fudan University, Shanghai, China; Cornell University, UNITED STATES

## Abstract

Meiotic recombination is required for proper homologous chromosome segregation in plants and other eukaryotes. The eukaryotic *RAD51* gene family has seven ancient paralogs with important roles in mitotic and meiotic recombination. Mutations in mammalian *RAD51* homologs *RAD51C* and *XRCC3* lead to embryonic lethality. In the model plant *Arabidopsis thaliana*, *RAD51C* and *XRCC3* homologs are not essential for vegetative development but are each required for somatic and meiotic recombination, but the mechanism of *RAD51C* and *XRCC3* in meiotic recombination is unclear. The non-lethal *Arabidopsis rad51c* and *xrcc3* null mutants provide an opportunity to study their meiotic functions. Here, we show that AtRAD51C and AtXRCC3 are components of the RAD51-dependent meiotic recombination pathway and required for normal AtRAD51 localization on meiotic chromosomes. In addition, AtRAD51C interacts with both AtRAD51 and AtXRCC3 *in vitro* and *in vivo*, suggesting that these proteins form a complex (es). Comparison of AtRAD51 foci in meiocytes from *atrad51*, *atrad51c*, and *atxrcc3* single, double and triple heterozygous mutants further supports an interaction between AtRAD51C and AtXRCC3 that enhances AtRAD51 localization. Moreover, *atrad51c*^*-/+*^ *atxrcc3*^*-/+*^ double and *atrad51*^*-/+*^ *atrad51c*^*-/+*^ *atxrcc3*^*-/+*^ triple heterozygous mutants have defects in meiotic recombination, suggesting the role of the AtRAD51C-AtXRCC3 complex in meiotic recombination is in part AtRAD51-dependent. Together, our results support a model in which direct interactions between the RAD51C-XRCC3 complex and RAD51 facilitate RAD51 localization on meiotic chromosomes and RAD51-dependent meiotic recombination. Finally, we hypothesize that maintenance of RAD51 function facilitated by the RAD51C-XRCC3 complex could be highly conserved in eukaryotes.

## Author summary

Meiotic recombination and sister chromatid cohesion are important for maintaining the association between homologous chromosomes and ensuring their accurate segregation. Meiotic recombination starts with a set of programmed DNA double-strand breaks (DSBs), catalyzed by the SPO11 endonuclease. Processing of DSB ends produces 3′ single-stranded DNA tails, which form nucleoprotein filaments with RAD51 and DMC1, homologs of the prokaryotic RecA protein. The eukaryotic *RAD51* gene family has seven ancient paralogs, in addition to RAD51 and DMC1, the other five members in mammals form two complexes: RAD51B-RAD51C-RAD51D- XRCC2 (BCDX2) and RAD51C-XRCC3 (CX3). To date, the molecular mechanism of CX3 in animal meiosis remains largely unknown due to the essential roles of these two proteins in embryo development. In Arabidopsis, RAD51C and XRCC3 are required for meiosis and fertility, but their specific mechanisms are unclear. Here we present strong evidence that *Arabidopsis* RAD51 forms a protein complex with AtRAD51C-AtXRCC3 *in vivo*. Our data also support the previous hypothesis that CX3 promotes RAD51-denpendet meiotic recombination by affecting its localization on chromosomes. Given that the RAD51, RAD51C and XRCC3 proteins are highly conserved in plants and vertebrates, the mechanism we present here could be important for the regulation of meiotic recombination in both plants and vertebrate animals.

## Introduction

Homologous recombination (HR) is important for repairing DNA damage and maintaining genomic stability. Meiotic HR and sister chromatid cohesion are required for maintaining physical associations between homologous chromosomes (homologs) and ensuring their accurate segregation. Meiotic HR is initiated by programmed DNA double-strand breaks (DSBs) that are catalyzed by SPO11, a topoisomerase-like protein [1]. The resulting DSB ends are processed by the MRE11- RAD50-NBS1 (MRN) protein complexes to generate 3′ single-stranded DNA (ssDNA) tails [2,3], which are subsequently protected by replication protein A (RPA) [4]. Functional homologs of the *E*. *coli* RecA protein, RAD51 and DMC1 [5,6] bind to the 3ʹ tails to form nucleoprotein filaments with the help of several proteins identified in multiple species, including *Saccharomyces cerevisiae* (Rad52 [7], Rad54 [8], Tid1/Rhd54 [9], Rad55-Rad57 [10], Swi5-Sfr1 [11] and PCSS complex [12]), *Arabidopsis thaliana* (RAD51C [13], XRCC3 [14], MND1-HOP2[15] and ATR/ATRIP [16]), and mammals (Mnd1-Hop2 [17] and Brca2-Dss1 [18]). The nucleoprotein filaments facilitate single-end invasion of a non-sister chromatid, resulting in the formation of a recombination intermediate called a D-loop, which is then processed to ultimately produce either crossovers (COs) or non-crossovers (NCOs) [19].

In vertebrate animals and plants, the *RAD51* gene family is highly conserved with seven members: *DMC1*, *RAD51*, *RAD51B*, *RAD51C*, *RAD51D*, *XRCC2* and *XRCC3* [20–23], which share Walker A and Walker B motifs with over 37.5% similarity [24]. In mice, mutations in any of the paralogs, except *DMC1*, lead to embryonic lethality following spontaneous DNA damage or errors [25–29]. In the model plant *Arabidopsis thaliana*, all seven genes are dispensable for vegetative growth [13,14,24,30–33]. However, *AtRAD51*, *AtRAD51C* and *AtXRCC3* are required for somatic and meiotic recombination, as well as plant fertility. Mutations in any of these three genes result in a meiotic chromosome fragmentation phenotype [13,14,24,30–32]. Moreover, *AtDMC1* is specifically required for meiotic homolog pairing and recombination [34,35]. In contrast to *atrad51*, *atrad51c* and *atxrcc3* mutants, *atdmc1* mutants do not suffer meiotic chromosome fragmentation; instead their DSBs are thought to be repaired using sister chromatids as templates [34,35]. The three other paralogs, *AtRAD51B*, *AtRAD51D* and *AtXRCC2*, seem to be unnecessary for meiotic DSB repair, because the triple mutant has normal chromosome morphology and fertility [33]. Except for slight differences in synapsis, the chromosome morphology using light microscopy for DAPI-stained chromosomes and fertility phenotypes of *atrad51c* and *atxrcc3* mutants are similar to those of *atrad51*, suggesting that their functions are related, but further analyses are needed to understand their mechanistic roles in meiotic DSB repair.

Biochemical studies in human cells demonstrate that RAD51 paralogs associate with one another in two distinct complexes: RAD51B-RAD51C-RAD51D-XRCC2 (BCDX2) and RAD51C-XRCC3 (CX3) [36,37]. The CX3 complex stabilizes RAD51 binding to ssDNA [36–39] *in vitro*, thus promoting single-end invasion. Moreover, RAD51C and XRCC3 also help mediate Holliday junction (HJ) resolution *in vitro* [40], suggesting a later role in meiotic recombination. A yeast two-hybrid assay demonstrated that the *Arabidopsis* RAD51 paralogs also interact with each other [41], supporting the idea that RAD51 paralogs function by formation of distinct protein complexes in both animals and plants. However, whether the RAD51 paralogs associate with each other *in planta* has not been tested.

In this study, we report that *Arabidopsis* homologs of RAD51, RAD51C and XRCC3 show highly similar meiotic chromosome morphological defects using immune-localization for key markers. We also provide evidence that AtRAD51C and AtXRCC3 are required for AtRAD51 localization on chromosomes. Both *in vitro* and *in vivo* data demonstrate that AtRAD51C interacts with AtRAD51 and AtXRCC3. Furthermore, observation of AtRAD51 foci in *atrad51*, *atrad51c* and *atxrcc3* single, double and triple heterozygotes reveals that AtRAD51C and AtXRCC3 both are involved in AtRAD51 loading. Triple heterozygotes also experience non-homolog chromosome associations and have reduced CO frequencies. Together, these results demonstrate that AtRAD51C, AtXRCC3 and AtRAD51 form a complex *in planta* and are required for AtRAD51 loading on chromosomes.

## Results

### *AtRAD51*, *AtRAD51C* and *AtXRCC3* have non-redundant roles in meiotic recombination

Previous studies have found that *AtRAD51*, *AtRAD51C* and *AtXRCC3* are required for meiotic DSB repair and plant fertility and mutation of individual genes cause indistinguishable chromosome entanglement and fragmentation phenotypes [13,14,31,32]. The similarity of the phenotypes suggests that these genes might function in the same genetic pathway or process. To test this hypothesis, we generated double mutants between *atrad51-3* (SAIL_873_C08) [42], *atrad51c* (SALK_021960) [13], and *atxrcc3* (SALK_045564) [14] and found that the chromosome morphologies of *atrad51 atrad51c* (48 cells), *atrad51 atxrcc3* (65 cells), and *atrad51c atxrcc3* (54 cells) double mutants showed no obvious differences compared with each of the single mutants (S1 Fig). The lack of an additive phenotype in the double mutants further supports the hypothesis that they act together in the same biological process.

**Fig 1:**
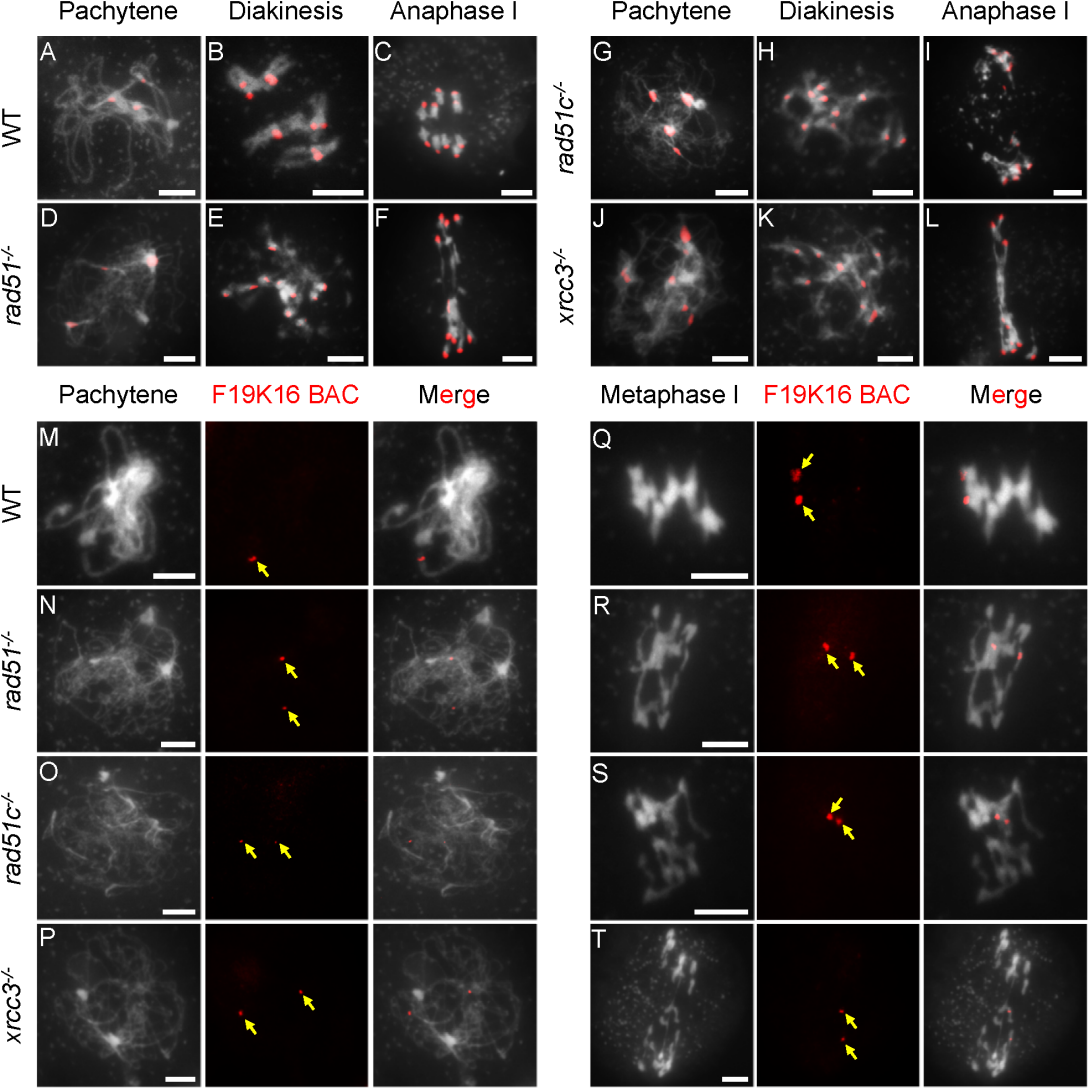
Fluorescence *in situ* hybridization (FISH) analysis of chromosome interactions in *atrad51*, *atrad51c* and *atxrcc3* mutants. (A-L) Chromosome morphologies of wild type (WT), *atrad51*, *atrad51c* and *atxrcc3* mutants at pachytene, diakinesis and anaphase I. Centromeres appear as red dots. (M-P) WT pachytene chromosomes showing a single signal from the BAC-F19K16 probe and *atrad51*, *atrad51c*, and *atxrcc3* chromosomes showing two signals from the same probe. (Q-S) Equal segregation of BAC-F19K16 signals at metaphase I in WT and unequal segregation in *atrad51* and *atrad51c* mutants. (T) BAC-F19K16 signals located on *atxrcc3* chromosome fragments at metaphase I. For FISH analysis of chromosomes using the BAC-F19K16 probe, the left panels show the chromosome morphology following staining with 6-diamidino-2-phenylindole (DAPI), the middle panels show the BAC-F19K16 signals (red dots) and the right panels merge the DAPI staining and BAC-F19K16 signals. Scale bar: 5 μm.

To search for subtle chromosomal phenotypes that could discriminate between the three mutants, we used FISH with a centromere probe for *atrad51* (82 cells); *atrad51c* (96 cells) and *atxrcc3* (81cells) and a bacterial artificial chromosome (BAC-F19K16) probe that targets a telomere proximal region on chromosome 1 for *atrad51* (31 cells); *atrad51c* (45 cells) and *atxrcc3* (22 cells) [43]. Wild-type (WT) meiocytes had three to five centromere signals at pachytene, indicative of paired homologous centromeres in a cluster (Fig 1A). Although the three mutants had no typical pachytene chromosomes, they all displayed similar centromere clusters or numbers of signals at a stage similar to that of WT, suggesting that *AtRAD51*, *AtRAD51C* and *AtXRCC3* are not required for early centromere pairing or clustering (Fig 1D, 1G and 1J). At diakinesis and metaphase I, WT meiocytes had five bivalents, each with two paired centromere signals corresponding to the associated homologs (Fig 1B). In contrast, the three mutants each had 10 centromere signals located on abnormally associated chromosomes (multivalents-with more than two chromosomes) (Fig 1E, 1H and 1K), indicating a failure to maintain homolog association, at least at the centromere regions. We next examined homolog pairing on the chromosome arms using the telomere-proximal BAC probe. Unlike the single focus observed on WT pachytene chromosomes, indicative of fully synapsed homologs, meiocytes from each of the three mutants showed two separate signals, indicating a failure to pair properly (Fig 1M–1P). We also performed ASY1 and ZYP1 immuno-localization in WT and mutants. No obvious difference of ASY1 signals at zygotene was found between WT and mutants (S2 Fig). However, unlike WT with linear ZYP1 distribution on pachytene chromosomes, ZYP1 was completely disappeared in *rad51*, while some punctate or discontinuous ZYP1 signals were observed in *xrcc3* and *rad51c* (S2 Fig). Together, these results demonstrate that *AtRAD51*, *AtRAD51C* and *AtXRCC3* are not required for recombination-independent centromere clustering, but are necessary for homolog pairing, consistent with previous findings obtained using FISH experiment [44]. The similarities of the mutant phenotypes further support the idea that they act in the same process.

### *Arabidopsis* RAD51C and XRCC3 are required for normal localization of RAD51 on chromosomes

**Fig 2:**
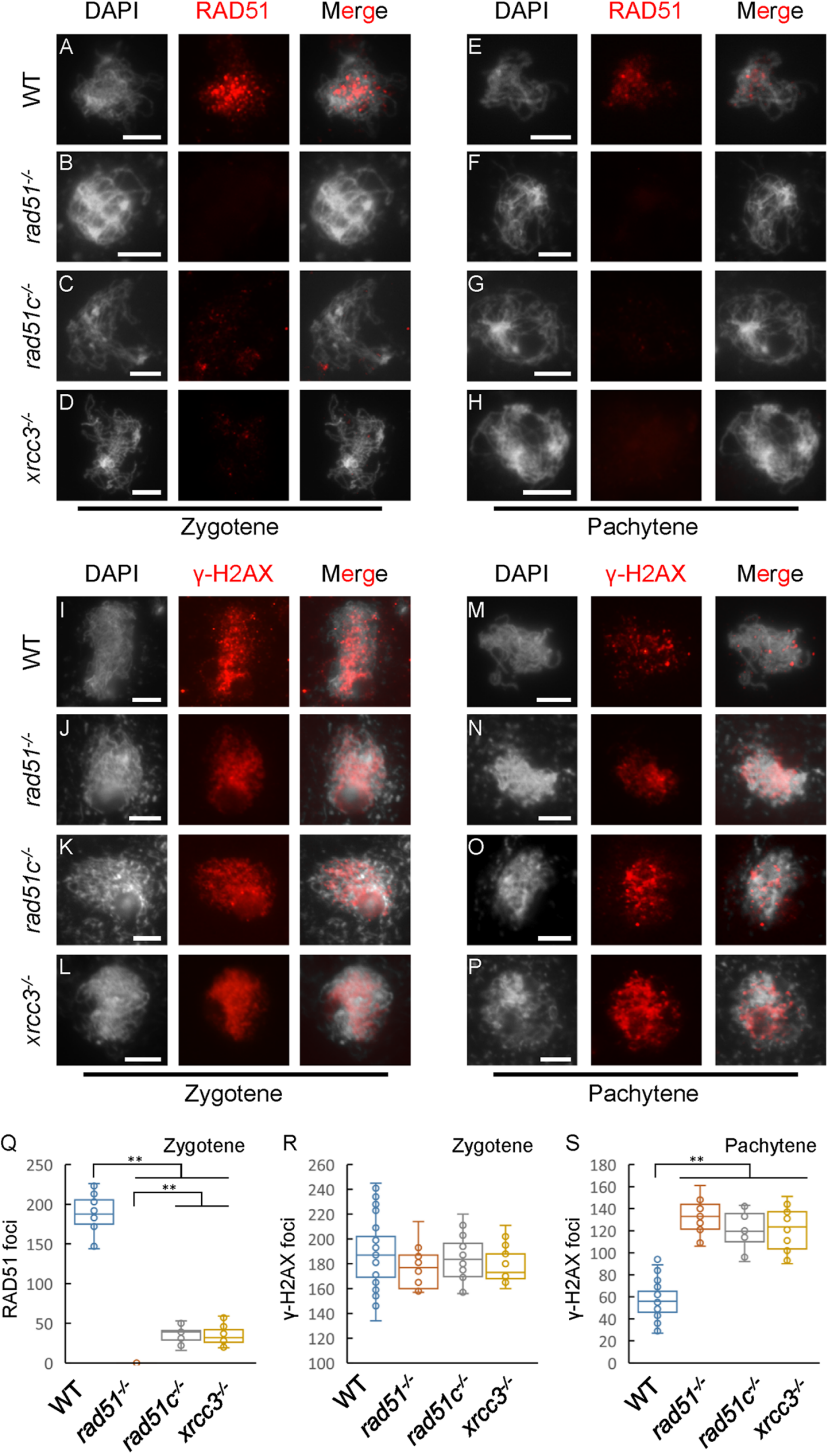
Immunofluorescence of AtRAD51 and γ-H2AX in wild type, *atrad51*, *atrad51c* and *atxrcc3* mutants. (A, E) Localization of RAD51 on wild-type (WT) chromosomes at zygotene and pachytene stages, respectively. (B, F) No signal was detected in the *atrad51* mutant at similar stages. (C, D, G, H) The numbers of AtRAD51 foci were significantly reduced in the *atrad51c* and *atxrcc3* mutants compared with WT at zygotene and pachytene, respectively. (I, M) Localization of γ-H2AX on wild-type (WT) chromosomes at zygotene and pachytene stages, respectively. (J-L) No significant differences were detected between *atrad51*, *atrad51c* and *atxrcc3* mutants at zygotene. (N-P) More γ-H2AX foci were detected at pachytene in *atrad51*, *atrad51c* and *atxrcc3* mutants compared to WT. (Q) The number of RAD51 foci on chromosomes from wild type and mutants at zygotene. (R-S) The number of γ-H2AX foci on chromosomes from wild type and mutants at zygotene and pachytene. Left panels show the chromosome morphology following staining with 6-diamidino-2-phenylindole (DAPI), middle panels show AtRAD51 foci (red dots), and right panels merge the DAPI-stained images with the AtRAD51 foci images. Scale bar: 5 μm. ** p<0.01 (two-tailed Student’s *t*-test).

Loading of RAD51 on ssDNA is aided by several proteins, including Rad52 [45], Rad55-57 (Rad51 paralogs) [46] and Sfr1-Swi5 [11] in yeast, the Brca2-Dss1 complex in mammalian cells [47], and also by AtBRCA2 in *Arabidopsis* [48]. The similarity of meiotic defects in *Arabidopsis rad51*, *rad51c* and *xrcc3* mutants suggests the RAD51 paralogs RAD51C and XRCC3 may function in meiotic recombination by affecting RAD51 function. To test this hypothesis, we performed an immunofluorescence assay using an AtRAD51 antibody [49]. In *Arabidopsis*, formation of DSBs is thought to occur at leptotene [50]. At a similar stage, we found that WT plants had 187.7±24.5 AtRAD51 foci per meiocyte (n = 14), but the number of foci was greatly reduced in *atrad51c* (36.1±9.7, n = 17; P = 1.5E-13) and *atxrcc3* (33.7±10.3, n = 34; P = 5.7E-13) mutant meiocytes (Fig 2A, 2C, 2D and 2Q). In contrast, a parallel experiment did not detect any AtRAD51 foci in *atrad51* mutant meiocytes at zygotene (Fig 2B). A similar pattern was also observed using pachytene meiocytes (Fig 2E–2H). These results provide evidence that *Arabidopsis* RAD51C and XRCC3 are required for formation of wild type level of RAD51 foci on meiotic chromosomes. This is consistent with the previous findings for Rad51 paralogs in yeast [46]. Nevertheless, the reduction of AtRAD51 foci in *atrad51c* and *atxrcc3* homozygous mutants does not preclude the possibility that normal level DSBs are formed in these mutants. To test whether DSB frequency is altered in *atrad51c* and *atxrcc3* mutants, we examined the distribution of a DSB marker, phosphorylated histone H2AX (γ-H2AX) [51]. At zygotene, after DSBs have been formed, no significant differences in the number of γ-H2AX foci were detected between WT (189.3±26.5, n = 39), *atrad51* (176.7±15.5, n = 19; P = 0.062), *atrad51c* (183.6±18.0, n = 18; P = 0.41) and *atxrcc3* (178.3±13.5, n = 19; P = 0.097) mutants (Fig 2I–2L and 2R). In *Arabidopsis*, most meiotic DSBs are thought to be repaired during zygotene-pachytene. We found that γ-H2AX foci were obviously reduced in WT (56.9±15.2, n = 55) pachytene meiocytes compared those of *atrad51* (132.1±15.4, n = 13; P = 1.5E-11), *atrad51c* (120.6±16.6, n = 14; P = 7.2E-11) and *atxrcc3* (122.2±18.8, n = 18; P = 1.7E-11) mutants (Fig 2M–2P and 2S). The presence of normal numbers of zygotene γ-H2AX foci and reduced AtRAD51 foci suggests that AtRAD51C and AtXRCC3 are not required for meiotic DSB formation, but are necessary for AtRAD51 loading.

### *Arabidopsis* RAD51C interacts with RAD51 and XRCC3 *in vitro* and *in vivo*

In yeast, the Rad51 paralogs Rad55 and Rad57 form a heterodimeric complex to stimulate RAD51 activity [10]. Vertebrate Rad51 paralogs interact with one another to form two distinct complexes: BCDX2 and CX3 [52]. Like vertebrates, *Arabidopsis* has seven RAD51 paralogs, and previous yeast two-hybrid assays have shown that XRCC3 interacts with both RAD51C and RAD51 [41]. However, whether these proteins interact *in planta* has not been investigated.

**Fig 3:**
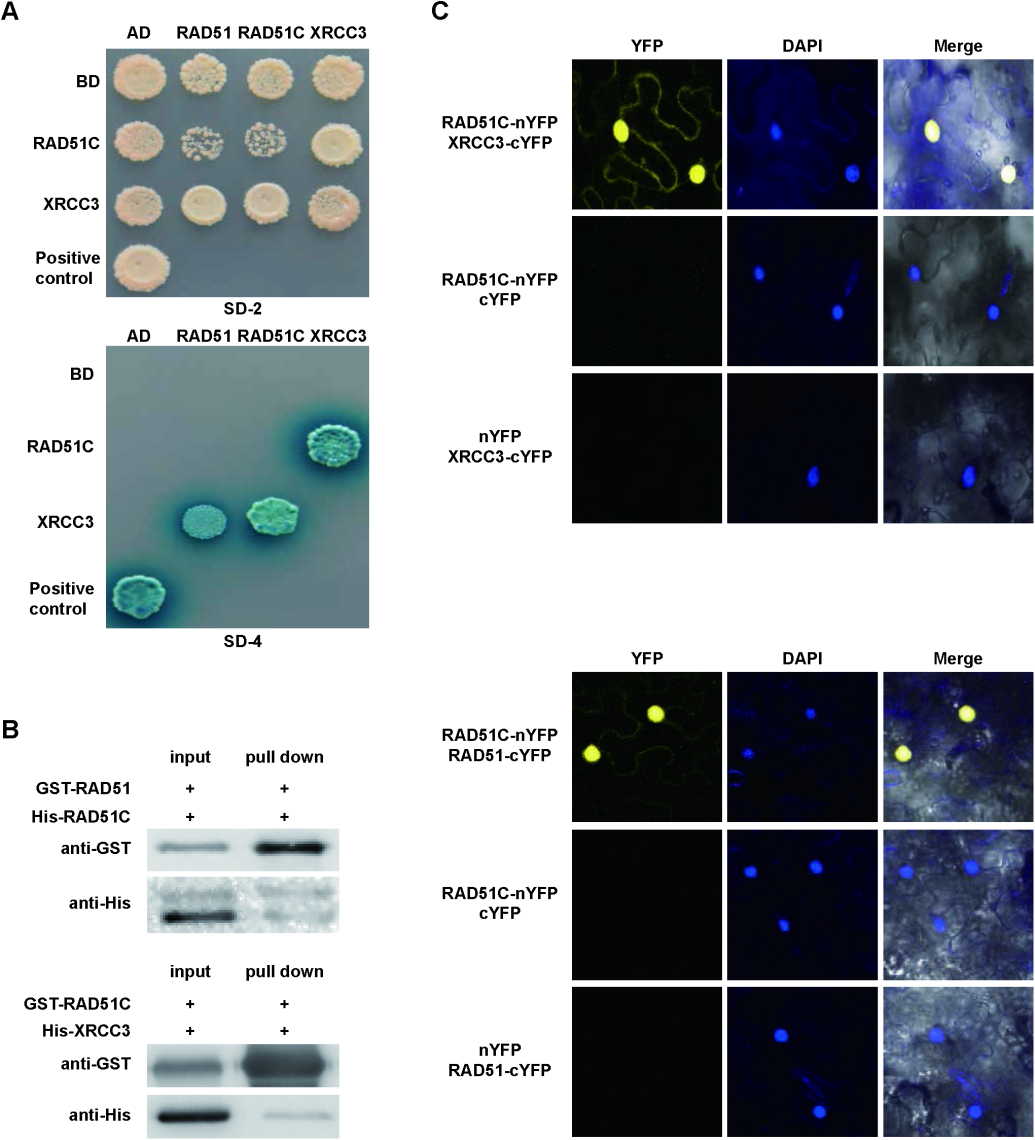
AtRAD51C interacts with AtRAD51 and AtXRCC3 *in vitro* and *in vivo*. (A) Yeast two-hybrid assay showing AtXRCC3 interaction with AtRAD51 and AtRAD51C. The known DYT1-DYT1 interaction is used as a positive control [82]. AD refers to the activation domain, BD refers to the DNA-binding domain, SD-2 refers to SD-Leu-Trp and SD-4 refers to SD-Leu-Trp-His-Ade+X-gal. Blue indicates a positive interaction. (B) Pull-down assay showing that AtRAD51C interacts with AtRAD51 and AtXRCC3. (C) BiFC assay in tobacco cells showing strong nuclear signals for AtRAD51-AtRAD51C and AtRAD51C-AtXRCC3 interactions. Left panels are yellow fluorescent protein (YFP) signals, middle panels are nuclei stained with 6-diamidino-2-phenylindole (DAPI), and right panels merge the DAPI-stained images with the YFP signals and bright field images.

As an initial test for potential interactions we used a yeast two-hybrid assay (Y2H) and found that AtXRCC3 interacts with both AtRAD51 and AtRAD51C (Fig 3A), consistent with the previously identified interactions in Y2H system [41]. The interaction between AtRAD51C and AtXRCC3 was further supported by a pull-down assay using recombinant fusion protein of glutathione S-transferase (GST) with AtRAD51C and an AtXRCC3-His tag fusion protein (Fig 3B). In addition to the previously identified interactions, we also found that GST-AtRAD51 interacts with AtRAD51C-His (Fig 3B). To explore whether these associations also occurred *in planta*, we examined the interactions using bimolecular fluorescence complementation (BiFC) in tobacco (*Nicotiana benthamiana*) cells. Strong nuclear signals, indicating interaction, were observed for AtRAD51C with either AtRAD51 or AtXRCC3 (Fig 3C). These results provide the first direct evidence that plant RAD51 paralogs RAD51C and XRCC3 interact directly with RAD51 *in vitro* and *in planta*.

### Male meiotic defects are observed in *atrad51c*^*-/+*^ *atxrcc3*^*-/+*^ double and *atrad51*^*-/+*^ *atrad51c*^*-/+*^ *atxrcc3*^*-/+*^ triple heterozygous mutants

A recent study identified a weak *atrad51* allele, *atrad51-2* [42], with a T-DNA insertion in the 3′-untranslated region (UTR) that results in reduced AtRAD51 protein levels. This mutant had mild chromosome fragmentation and partial synapsis, as well as some bivalent formation with homologs and non-homologs [42]. In contrast, the *atrad51-1* null mutant had severe chromosome fragmentation and formed multivalents during meiotic prophase I [31]. These findings suggest that reducing AtRAD51 level might be a strategy for investigating its meiotic function. Alternatively, analysis of double heterozygous mutants in genes encoding components of a complex can reveal phenotypic defects, even though the corresponding single heterozygotes are phenotypically normal [53,54]. We hypothesized that double/triple heterozygotes of *atrad51*, *atrad51c* and *atxrcc3* might reduce, but not abolish, their interactions in a complex and reveal informative meiotic phenotypes

**Fig 4:**
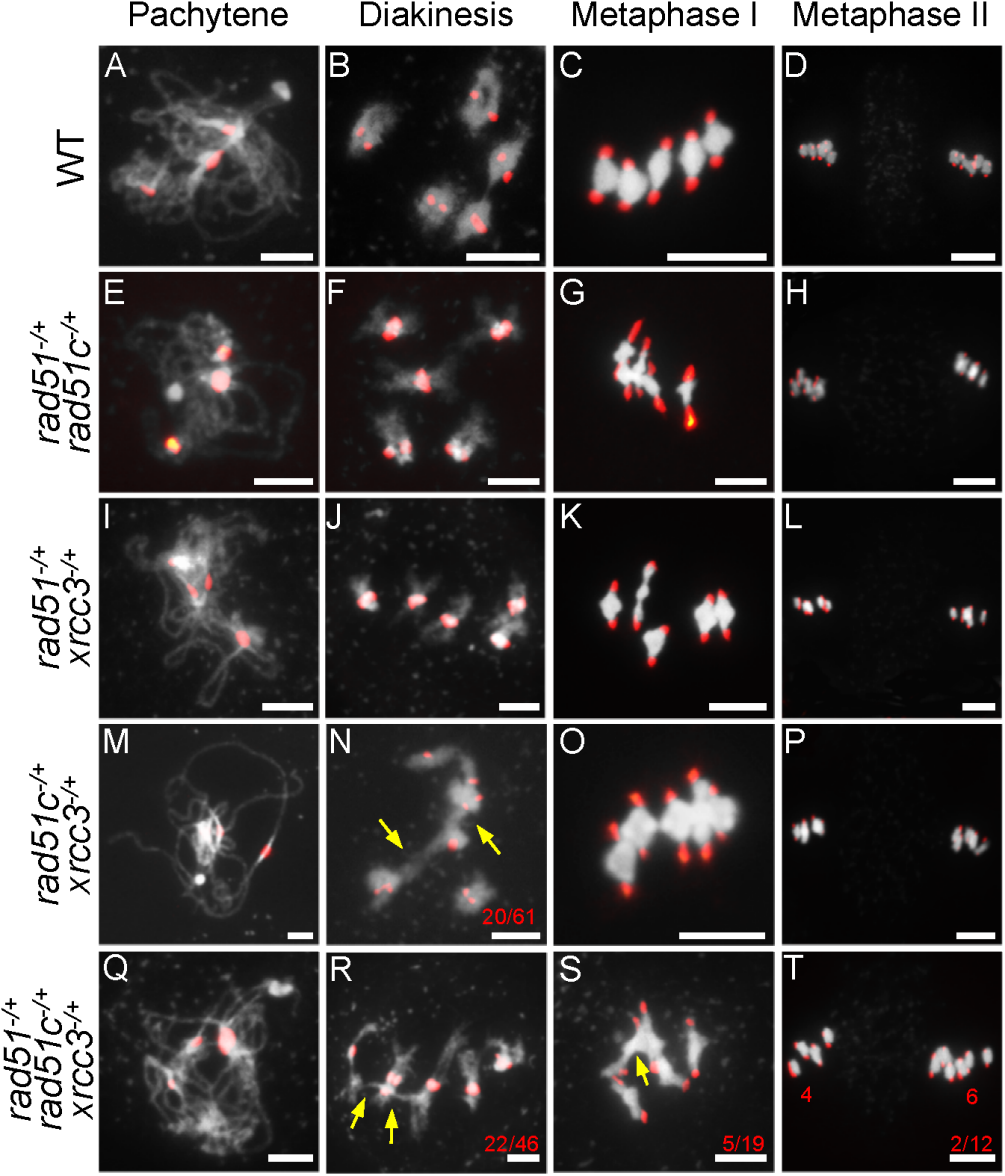
Centromere-fluorescence *in situ* hybridization analysis of chromosome morphology in double and triple heterozygous mutants. (A-L) Wild type (WT), *atrad51*^*-/+*^ *atrad51c*^*-/+*^ and *atrad51*^*-/+*^ *atxrcc3*^*-/+*^ mutant chromosome morphologies at pachytene, diakinesis, metaphase I and metaphase II. Compared with WT, *atrad51*^*-/+*^ *atrad51c*^*-/+*^ and *atrad51*^*-/+*^ *atxrcc3*^*-/+*^ mutants had similar chromosome morphologies. (M) *atrad51c*^*-/+*^ *atxrcc3*^*-/+*^ chromosome morphology at pachytene was similar to WT, but showed 32.8% (n = 61) non-homolog association at diakinesis (N). (O, P) *atrad51c*^*-/+*^ *atxrcc3*^*-/+*^ chromosome morphology at metaphase I and II, respectively. Chromosome morphology of *atrad51*^*-/+*^ *atrad51c*^*-/+*^ *atxrcc3*^*-/+*^ at pachytene (Q) and diakinesis (R), metaphase I (S) and metaphase II (T). The *atrad51*^*-/+*^ *atrad51c*^*-/+*^ *atxrcc3*^*-/+*^ triple chromosome morphology showed more severe defects than *atrad51c*^*-/+*^ *atxrcc3*^*-/+*^. Red dots indicate centromere signals. Yellow arrows show non-homologous chromosome associations (N, R, S). The red numbers in N, R, S refer to the number of abnormal cells out of all cells observed. Scale bar: 5 μm.

To test this hypothesis, we generated *atrad51*^*-/+*^, *atrad51c*^*-/+*^ and *atxrcc3*^*-/+*^ double and triple heterozygous mutants and compared their meiotic phenotypes with WT. Analysis of meiotic chromosome morphology after DAPI staining showed that *atrad51*^*-/+*^, *atrad51c*^*-/+*^ and *atxrcc3*^*-/+*^ single heterozygote meiocytes and *atrad51*^*-/+*^ *atrad51c*^*-/+*^ and *atrad51*^*-/+*^ *atxrcc3*^*-/+*^ double heterozygotes had similar phenotypes compared to WT (Fig 4A–4L; S3 Fig). In addition, meiocytes from *atrad51c*^*-/+*^ *atxrcc3*^*-/+*^ double heterozygotes had chromosome morphology similar to WT at pachytene (Fig 4M), but at diakinesis, WT formed five bivalents, whereas 32.8% (20 of 61, n = 61) of the *atrad51*^*-/+*^ *atxrcc3*^*-/+*^ double heterozygote meiocytes had non-homologous chromosome associations (Fig 4N). The cell appears to be able to resolve these associations since equal division of chromosomes was observed at anaphase I and II (Fig 4O and 4P). Meiocytes from *atrad51*^*-/+*^ *atrad51c*^*-/+*^ *atxrcc3*^*-/+*^ triple heterozygotes had a more severe non-homolog association phenotype (47.8% at diakinesis, 22 of 46, n = 46, Fig 4R) and had unequal chromosome segregation at metaphase II (16.7%, 2 of 12, n = 12, Fig 4T). No chromosome fragments were observed in the triple heterozygote, suggesting it is still capable of DSB repair. The results also suggest that RAD51C and XRCC3 are functionally more related to each other than either is to RAD51. Previous studies showed that T-DNA translocation can cause a similar pattern of chromosome association using light microscopy because the translocated chromosome can associated with two normal chromosomes [55,56]. We have verified the T-DNA insertion site by sequencing the junction with flanking genomic DNAs and the results indicated that these mutations are not associated with translocations.

### Repair of meiotic DSBs is delayed in *atrad51*^*-/+*^, *atrad51c*^*-/+*^ and *atxrcc3*^*-/+*^ single, double and triple heterozygous mutants

**Fig 5:**
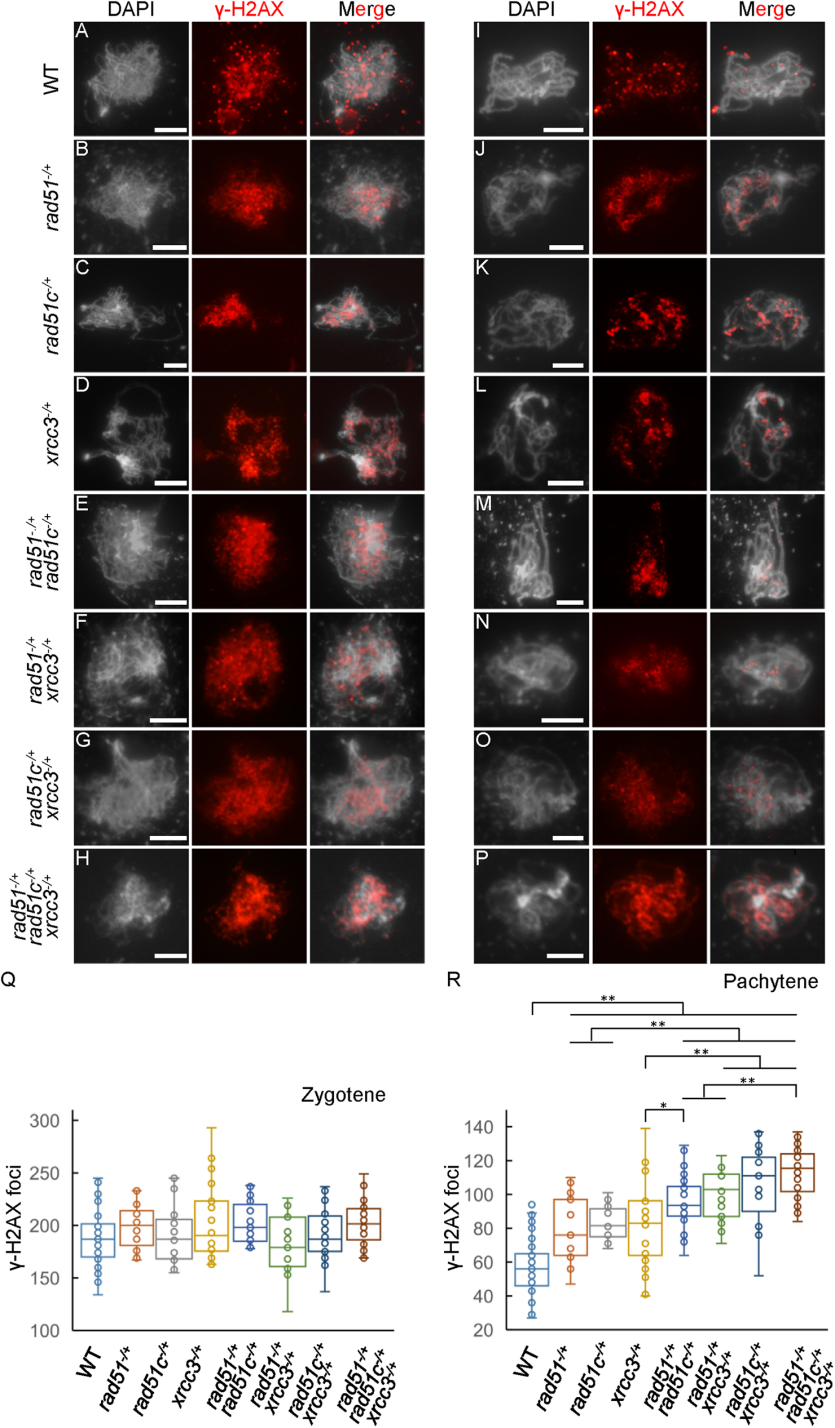
Immunostaining of γ-H2AX signals in single, double and triple heterozygous mutant chromosomes at zygotene and pachytene stages. (A-H) The distribution of γ-H2AX on zygotene chromosomes showed no obvious differences among the eight genotypes examined. (I-P) The γ-H2AX distribution in pachytene chromosomes from single, double and triple heterozygous mutants. Left panels show the chromosome morphology following staining with 6-diamidino-2-phenylindole (DAPI), middle panels show γ-H2AX foci (red dots), and right panels merge the DAPI-stained images with the γ-H2AX foci images. (Q-R) The number of γ-H2AX foci in chromosomes from the eight genotypes at zygotene and pachytene in A-P. Scale bar: 5 μm. * p<0.05, ** p<0.01 (two-tailed Student’s *t*-test).

To test whether meiotic DSB repair is delayed in the heterozygotes, we performed immunostaining experiments using a γH2AX antibody. As mentioned above, WT meiocytes had 189.3±26.5 (n = 39) and 56.9±15.2 (n = 55) γH2AX foci at zygotene and pachytene, respectively (Fig 5A, 5I, 5Q and 5R and Table 1). All single, double and triple heterozygotes had no obvious differences in the number of γH2AX foci at zygotene, but had significantly more foci at pachytene (Fig 5A–5R, S1 Table). Moreover, the double and triple heterozygotes had more foci at pachytene than the single heterozygotes. There are significantly fewer foci in *atrad51*^*-/+*^ (78.1±19.4, n = 17), *atrad51c*^*-/+*^ (83.3±10.8, n = 12) and *atxrcc3*^*-/+*^ (82.0±25.9, n = 24) (S1 Table) compared to the double mutants *atrad51*^*-/+*^ *atrad51c*^*-/+*^ (96.3±15.4, n = 30), *atrad51*^*-/+*^*atxrcc3*^*-/+*^ (100.4±14.8, n = 21) and *atrad51c*^*-/+*^*atxrcc3*^*-/+*^ (105.2±24.1, n = 15), which in turn have significantly fewer foci (S1 Table) than the triple *atrad51*^*-/+*^ *atrad51c*^*-/+*^ *atxrcc3*^*-/+*^ (113.3±14.8, n = 46) (Fig 5J–5P and 5R and Table 1). These data suggest that DSB formation is normal in the heterozygotes, but there is a defect in the progression of DSB repair, and that AtRAD51, AtRAD51C and AtXRCC3 function in this process.

**Table 1:** Numbers of γ-H2AX and AtRAD51 foci in single, double and triple heterozygous mutants at zygotene and pachytene.

	AtRAD51		γ-H2AX	
Genetype	Zygotene	Pachytene	Zygotene	Pachytene
**WT**	187.7±24.5 (n = 14)	51.2±14.0 (n = 65)	189.3±26.5 (n = 39)	56.9±15.2 (n = 55)
***atrad51***^***-/+***^	143.3±20.2 (n = 16)	49.8±13.2 (n = 25)	199.5±20.6 (n = 22)	78.1±19.4 (n = 17)
***atrad51c***^***-/+***^	143.8±32.7 (n = 22)	57.3±10.4 (n = 12)	191.0±28.3 (n = 30)	83.3±10.8 (n = 12)
***atxrcc3***^***-/+***^	142.2±29.7 (n = 19)	49.3±14.7 (n = 29)	201.9±35.4 (n = 24)	82.0±25.9 (n = 24)
***atrad51***^***-/+***^ ***atrad51c***^***-/+***^	116.4±23.1 (n = 12)	34.4±10.2 (n = 29)	202.9±20.2 (n = 19)	96.3±15.4 (n = 30)
***atrad51***^***-/+***^ ***atxrcc3***^***-/+***^	108.1±8.9 (n = 14)	35.5±9.8 (n = 35)	187.4±29.0 (n = 21)	100.4±14.8 (n = 21)
***atrad51c***^***-/+***^ ***atxrcc3***^***-/+***^	106.9±8.2 (n = 10)	32.2±9.0 (n = 33)	192.2±24.8 (n = 30)	105.2±24.1 (n = 15)
***atrad51***^***-/+***^ ***atrad51c***^***-/+***^ ***atxrcc3***^***-/+***^	99.5±13.0 (n = 18)	30.7±8.1 (n = 61)	199.5±16.3 (n = 35)	113.3±14.8 (n = 46)

**Fig 6:**
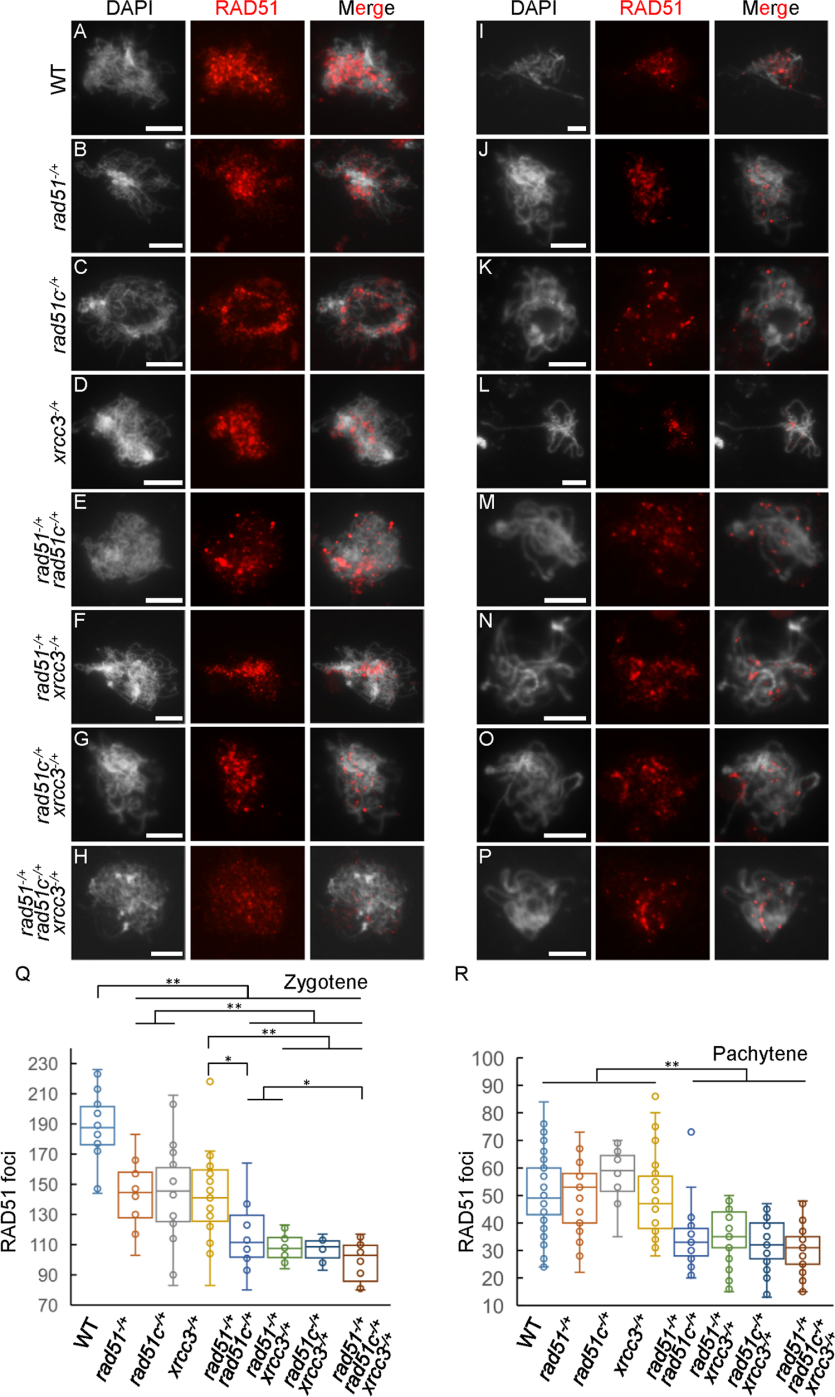
Immunostaining of AtRAD51 signals in single, double and triple heterozygous mutant chromosomes at zygotene and pachytene. (A-H) The locations and (Q) numbers of AtRAD51 foci on zygotene chromosomes from *atrad51*^*-/+*^, *atrad51c*^*-/+*^, *atxrcc3*^*-/+*^, *atrad51*^*-/+*^ *atrad51c*^*-/+*^, *atrad51*^*-/+*^ *atxrcc3*^*-/+*^, *atrad51c*^*-/+*^ *atxrcc3*^*-/+*^ and *atrad51*^*-/+*^ *atrad51c*^*-/+*^ *atxrcc3*^*-/+*^ heterozygous mutant meiocytes showed reductions compared to wild type (WT). (I-L) The location and (R) number of AtRAD51 foci on pachytene chromosomes in the three single heterozygotes showed no obvious differences compared with WT. (M-P) The location and (R) number of AtRAD51 foci on pachytene chromosomes in the double and triple heterozygous mutants were significantly reduced relative to WT. Left panels show the chromosome morphology following staining with 6-diamidino-2-phenylindole (DAPI), middle panels show AtRAD51 foci (red dots), and right panels merge the DAPI-stained images with the AtRAD51 foci images. Scale bar: 5 μm. ** p<0.01 (two-tailed Student’s *t*-test).

Because AtRAD51C and AtXRCC3 are required for normal AtRAD51 localization, we next examined AtRAD51 localization in heterozygous mutant meiocytes. As described above, WT meiocytes have 187.7±24.5 (n = 14) AtRAD51 foci at zygotene and 51.2±14.0 (n = 65) foci at pachytene (see Fig 6A and 6I for examples and Table 1). In contrast, single, double and triple heterozygous mutant meiocytes have significantly fewer AtRAD51 foci at zygotene (p<0.05; Fig 6A–6H and 6Q). At pachytene, the three single mutant heterozygotes show no obvious differences in the number of AtRAD51 foci compared with WT (Fig 6I–6L and 6R), but the double and triple heterozygotes exhibited reduced AtRAD51 foci (p<0.05; Fig 6M–6P and 6R). These findings are consistent with the earlier results, suggesting that AtRAD51C and AtXRCC3 play related roles in AtRAD51 loading on chromosomes, likely in a protein complex.

### CO number is reduced in *atrad51*^*-/+*^ *atrad51c*^*-/+*^ *atxrcc3*^*-/+*^ triple heterozygotes

**Fig 7:**
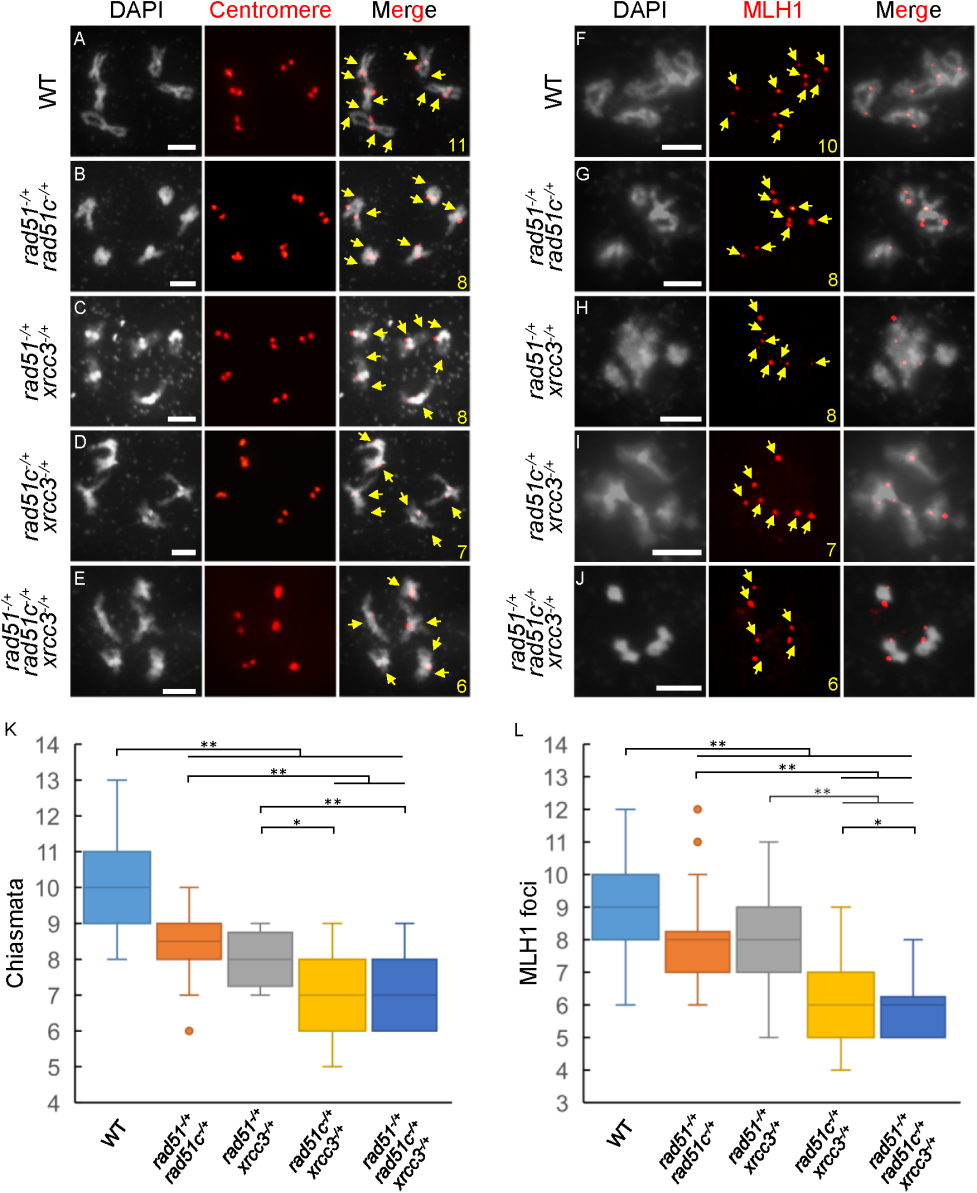
Number of chiasmata and AtMLH1 foci in double and triple heterozygous mutants. (A-E) Centromere-fluorescence *in situ* hybridization showing wild type (WT) and *atrad51*^*-/+*^ *atrad51c*^*-/+*^, *atrad51*^*-/+*^ *atxrcc3*^*-/+*^, *atrad51c*^*-/+*^ *atxrcc3*^*-/+*^ and *atrad51*^*-/+*^ *atrad51c*^*-/+*^ *atxrcc3*^*-/+*^ double and triple heterozygous mutant chromosome morphologies at diakinesis. Red dots indicate centromere signals. (F-J) Immunostaining of AtMLH1 foci (red dots) on WT, *atrad51*^*-/+*^ *atrad51c*^*-/+*^, *atrad51*^*-/+*^ *atxrcc3*^*-/+*^, *atrad51c*^*-/+*^ *atxrcc3*^*-/+*^ and *atrad51*^*-/+*^ *atrad51c*^*-/+*^ *atxrcc3*^*-/+*^ diakinesis chromosomes. (K) Number of chiasmata in WT, double and triple mutant chromosomes. (L) Number of AtMLH1 foci in WT, double and triple mutants. Scale bar: 5μm. * p<0.05, ** p<0.01 (one-tailed Student’s *t*-test).

As described earlier, the weak *atrad51-2* allele is capable of forming bivalents and executing recombination [42]. Similarly, the heterozygous plants analyzed here also completed meiotic recombination to some extent and had partial fertility. To examine CO frequencies in comparison between the various genotypes, we counted the number of chiasmata, the physical manifestation of crossing-over, in WT and mutant meiocytes at both diplotene and metaphase I. On average, WT had 10.1±1.1 (n = 52) chiasmata per meiocyte and no obvious significant differences were observed in the single heterozygotes: *atrad51*^*-/+*^ with 9.6±0.7 (n = 20; P = 0.072) per meiocyte, *atrad51c*^*-/+*^ with 9.6±0.7 (n = 21; P = 0.052) per meiocyte and *atxrcc3*^*-/+*^ with 9.6±0.8 (n = 24; P = 0.066) per meiocyte. The *atrad51*^*-/+*^ *atrad51c*^*-/+*^, *atrad51*^*-/+*^ *atxrcc3*^*-/+*^ and *atrad51c*^*-/+*^ *atxrcc3*^*-/+*^ double heterozygotes showed a slight, but statistically significant, reduction of chiasmata with 8.4±1.2 (n = 14; P = 6.0E-05), 8.0±0.8 (n = 10; P = 2.9E-06) and 7.1±1.0 (n = 34; P = 1.2E-20) per meiocyte, respectively (Fig 7A–7D and 7K). The *atrad51*^*-/+*^ *atrad51c*^*-/+*^ *atxrcc3*^*-/+*^ triple heterozygous mutant also had a significant reduction, with only 6.9±1.0 (n = 15; P = 2.6E-10) chiasmata per meiocyte formed (Fig 7E and 7K). Furthermore, the chiasmata numbers per meiocyte of *atrad51c*^*-/+*^ *atxrcc3*^*-/+*^ double heterozygote (7.1; P values = 2.0E-03 and 1.1E-02, respectively) and the triple heterozygote (6.9, P values = 1.7E-03 and 8.8E-03, respectively) were significantly lower than those of the other two double heterozygotes.

*Arabidopsis* forms two types of COs: interference-sensitive Type I COs that require ZMM proteins like MSH4 and MLH1 [57–59], and interference-insensitive class II COs that are MUS81-dependent [60,61]. To assess the impact of RAD51 and its paralogs on Type I COs, we used an AtMLH1 antibody to visualize AtMLH1 foci, in WT, *atrad51*^*-/+*^ *atrad51c*^*-/+*^, *atrad51*^*-/+*^ *atxrcc3*^*-/+*^, *atrad51c*^*-/+*^ *atxrcc3*^*-/+*^and *atrad51*^*-/+*^ *atrad51c*^*-/+*^ *atxrcc3*^*-/+*^ meiocytes at diakinesis [59]. On average, WT meiocytes had 9.0±1.2 foci (n = 61, Fig 7F), whereas at similar stages, *atrad51*^*-/+*^ *atrad51c*^*-/+*^, *atrad51*^*-/+*^ *atxrcc3*^*-/+*^, *atrad51c*^*-/+*^ *atxrcc3*^*-/+*^ and *atrad51*^*-/+*^ *atrad51c*^*-/+*^ *atxrcc3*^*-/+*^ mutants had 7.9±1.4 (n = 40; P = 5.8E-05), 7.7±1.6 (n = 25; P = 5.4E-04), 6.4±1.3 (n = 39; P = 5.9E-16) and 5.9±1.0 (n = 16; P = 5.5E-12) foci, respectively (Fig 7G–7J and 7L). The reduction of AtMLH1 foci in the mutants is consistent with the observed reduction in chiasmata, and supports the idea that Type-I COs are reduced in the mutants.

Although the CO number was obviously reduced by ~30% in the *atrad51*^*-/+*^ *atrad51c*^*-/+*^ *atxrcc3*^*-/+*^ triple heterozygote, no univalents were observed, consistent with a mechanism that ensures at least one CO per chromosome [62]. If the COs were distributed among the 5 *Arabidopsis* bivalents randomly, they would follow the Poisson function P (k COs per bivalent) = (λ^k^e^-λ^)/k! where λ is the mean number of COs per bivalent. Using this function, from the analyses of 52 WT and 15 *atrad51*^-/+^ *atrad51c*^-/+^ *atxrcc3*-^/+^ triple heterozygote meiocytes, we would expect to find 36 and 19 univalents in WT and the triple mutant, respectively, but none were observed (Table 2).

**Table 2:** Observed and expected nonexchange chromosomes.

Genotype	Chiasmata	P(k = 0)*	Meiocytes (n)	E0_exp_†	E0_obs_
WT	10	0.14	52	36	0
*atrad51*^-/+^ *atrad51c*^-/+^ *atxrcc3*^-/+^	7	0.25	15	19	0

*P(k) = (λ^k^e^-λ^)/k!

† E0_exp_ = P(k = 0) * n * 5 (number of bivalents per meiocyte)

**Fig 8:**
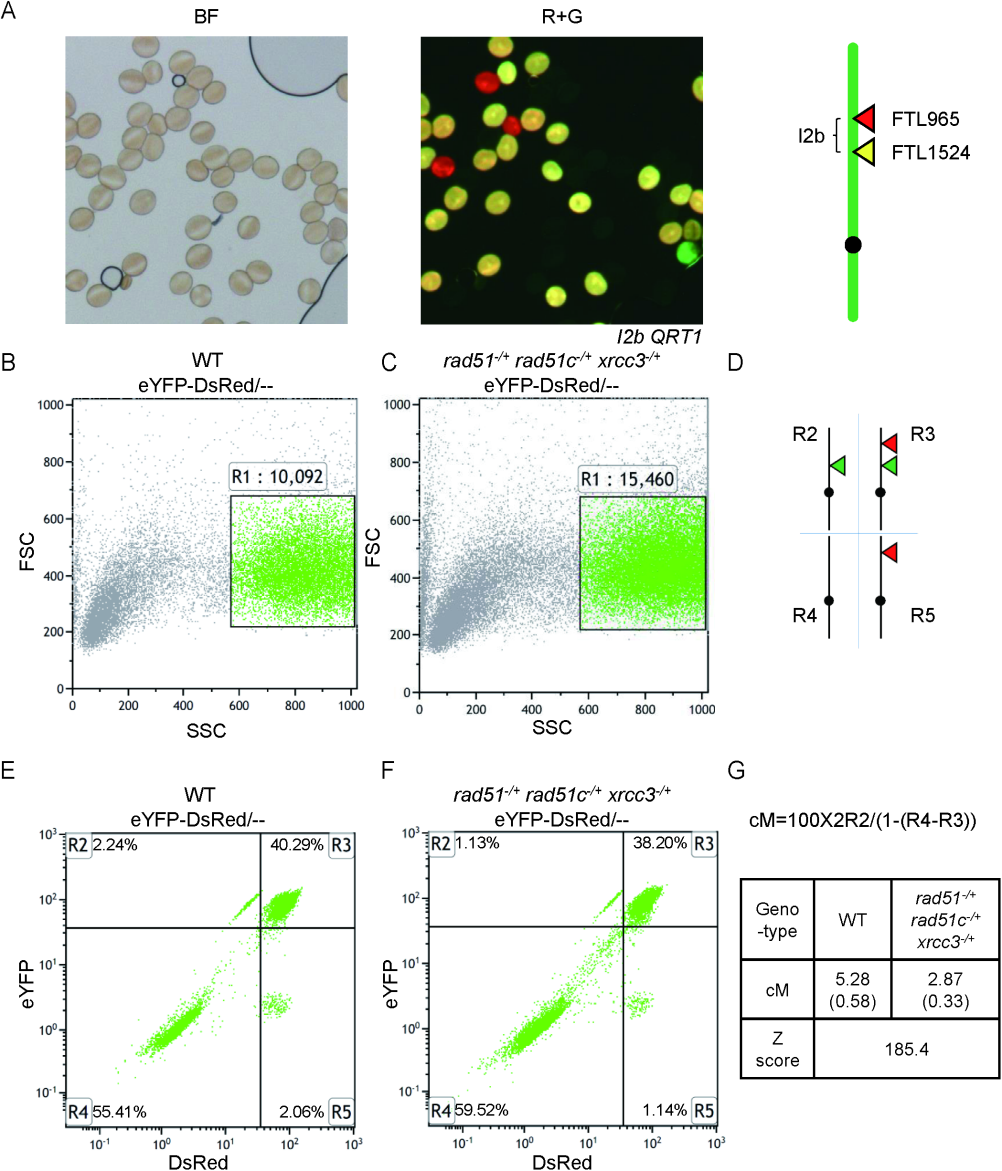
Meiotic crossover frequency of *I2b* in WT and triple heterozygote via flow cytometry. (A) Pollen grains from plants that are hemizygous for the DsRed2 (red grains) and eYFP (green pollen grains) markers flanking the *I2b* interval visualized using bright field (BF) microscopy or epi-fluorescence (R+G). Pollen grains expressing both markers are yellow. (B) Side scatter (SSC)/ forward scatter (FSC) plot of eYFP-DsRed/-- pollen grains (R1 gate) in WT with the number of pollen grains counted. Events in the R1 gate are represented as green points. (C) Side scatter (SSC)/ forward scatter (FSC) plot of eYFP-DsRed/-- pollen grains (R1 gate) in triple heterozygote with the number of pollen grains counted. Events in the R1 gate are represented as green points. (D) The R2 gate is comprised of yellow-only pollen grains; the R3 gate is comprised of the yellow+red pollen grains; the R4 gate R4 is comprised of non-fluorescent pollen grains; the R5 gate is comprised of red-only pollen grains. (E) DsRed / eYFP plot of eYFP-DsRed/-- pollen grains in WT with R2-R5 gates with the percentage of events in each quadrant is shown in each gate. (F) DsRed / eYFP plot of eYFP-DsRed/-- pollen grains in triple heterozygote with R2-R5 gates with the percentage of events in each quadrant is shown in each gate. (G) Formula to calculate the genetic distance of an interval flanked by the two fluorescent markers with results in WT and triple heterozygote. Standard deviations are shown in parentheses. Z score is 185.4: P value << 0.01.

To further quantify the remaining COs in *atrad51*^*-/+*^ *atrad51c*^*-/+*^ *atxrcc3*^*-/+*^, we used a flow cytometry-based assay that measures the segregation of transgenes encoding fluorescent marker proteins expressed using a pollen-specific LAT52 promoter (FTL markers) [63,64]. The number of viable pollen grains is dramatically reduced in *atrad51*^*-/+*^ *atrad51c*^*-/+*^ *atxrcc3*^*-/+*^, but it was still feasible to measure CO frequencies using this assay. We crossed *atrad51*^*-/+*^ *atrad51c*^*-/+*^ *atxrcc3*^*-/+*^ with line *I2b*, which carries two FTL markers (YFP and DsRed) on chromosome 2 (Fig 8A). Pollen grains which express both fluorescent proteins have not experienced a crossover between the markers, while those that express only one or the other have. The relative abundance of these two classes can be used to calculate the genetic distance between the two markers [65]. We scored 10,092 WT pollen grains and 15,460 pollen grains from the triple heterozygote (Fig 8B and 8C). The *I2b* map distance was 5.28±0.58 cM in WT and 2.87±0.33 cM in the triple heterozygote (Fig 8D–8G). The genetic distance between the two fluorescent markers was significantly reduced in *atrad51*^*-/+*^ *atrad51c*^*-/+*^ *atxrcc3*^*-/+*^ (Z score = 185.4, P value << 0.01) (Fig 8G), consistent with the reduction in chiasmata described above.

## Discussion

### Formation of protein complexes between RAD51 and its paralogs is highly conserved

RAD51 family members are conserved across species, from yeast to humans [20]. The budding yeast *S*. *cerevisiae* has four RAD51 paralogs (Rad51, Dmc1, Rad55 and Rad57) [10], whereas humans have seven paralogs (RAD51, DMC1, RAD51B, RAD51C, RAD51D, XRCC2, XRCC3) [20]. In yeast, Rad55 interacts with Rad57 to form a stable heterodimer [10]. Similarly, in humans, two complexes are formed by the RAD51 paralogs: the BCDX2 and the CX3 complexes [36,37,39]. Moreover, a recent study in *Caenorhabditis elegans* showed that the RAD51 paralogs, RFS-1 and RIP-1, also exist as a heterodimer and interact with RAD51 [66].

*Arabidopsis* XRCC3 has been shown to interact with both RAD51 and RAD51C using a yeast two-hybrid assay [41]. We confirmed the yeast two-hybrid result (Fig 3A) and demonstrated that the AtRAD51C-AtXRCC3 interaction occurs *in planta* by using pull-down and BiFC assays (Fig 3B and 3C). It is noteworthy that both pull-down and BiFC assays support an interaction between AtRAD51C with AtRAD51 and AtXRCC3. Our results strongly support the idea that AtRAD51C is a central factor in complex formation, and is associated with AtRAD51 and AtXRCC3. These findings are consistent with previous results in human cells that show AtRAD51C associates with two protein complexes [36,37,39]. Our study is also the first time to show that RAD51 paralogs form a protein complex with RAD51 in plants, supporting the hypothesis that formation of RAD51-paralogs associated protein complexes is highly conserved across eukaryotes, including yeast, humans and plants.

### Formation of a RAD51 paralog complex is required to facilitate RAD51 in HR

Previous studies in yeast showed that RAD51 paralogs are unable to form filaments with ssDNA and do not have a direct role in homology search or single strand invasion [10,23]. Nevertheless, studies in different organisms have reported that RAD51 paralogs play important roles in promoting RAD51 function in both mitotic and meiotic HR [46,67–69]. For example, the yeast Rad55-Rad57 complex has a role in RAD51-dependent HR [10,46]. Similar roles have been found for the *C*. *elegans* heterodimer of RAD51 paralogs RFS-1/RIP-1 [66] and the human CX3 complex [36,37,39].

Due to the lack of direct biochemical data, the role of RAD51 paralogs in meiotic HR *in planta* is unclear. Studies in the monocot model plant, *Oryza sativa* (rice), showed that the RAD51 paralogs OsRAD51C and OsXRCC3 are required for meiotic DSB repair and mutations in either result in sterility, chromosome entanglement and fragmentation [70,71]. These results are consistent with similar findings in *Arabidopsis* [13,14,32]. Immunostaining showed that OsXRCC3 is required for OsRAD51C localization on chromosomes [70], suggesting the existence of a potential OsRAD51C-OsXRCC3 complex in rice. Additionally, the single-end processing proteins OsCOM1 and OsDMC1 no longer associate with DSB sites in rice *osxrcc3*, which suggests that OsXRCC3, and by extension OsRAD51C, might function upstream of OsRAD51 [70,72]. Nevertheless, the relationship between OsRAD51 and its paralogs OsRAD51C and OsXRCC3 remains unclear, because a RAD51 antibody is currently unavailable in rice. In the present study, we showed that *Arabidopsis* RAD51 foci were obviously reduced in *atrad51c* and *atxrcc3* mutants, consistent to the discovery in rice. Together, these studies, in both rice and *Arabidopsis*, strengthen the idea that AtRAD51 depends on its paralogs for normal function and that this relationship is highly conserved in eukaryotes.

### RAD51 paralogs have a role in meiotic CO formation

Previous studies showed that RAD51 paralogs have a later role in processing meiotic recombination intermediates [40]. Direct evidence to support the RAD51C-XRCC3 complex having a role in the later meiotic recombination process come from the observation that the RAD51C-XRCC3 complex is associated with HJ resolvase activity. Moreover, RAD51C- and XRCC3-defective hamster cells have reduced resolvase activity and HJ progression [40,73]. Similarly, the *Arabidopsis* RAD51 paralogs AtRAD51B and AtXRCC2 were also reported to affect meiotic recombination in terms of CO number [74]. However, mutations in these paralogs show an increase in meiotic recombination frequency [74], suggesting that they have roles in meiotic CO formation. In the present study, we found that *atrad51c atxrcc3* double heterozygous mutant and the *atrad51 atrad51c atxrcc3* triple heterozygous mutant have significantly fewer COs (Fig 7D, 7E and 7I–7L), compared with WT. Given that the reduced number of AtRAD51 foci observed in the double and triple heterozygous mutants, we propose that a diminished capacity to form wild type level of RAD51 foci results in fewer COs in the mutants. The previous finding further supports this idea that a weaker *atrad51* allele had fewer chromosome fragments and some univalents, and also formed bivalents between homologs and non-homologs [42]. Therefore, we speculate that AtRAD51 could function in two manners, both dependent on the AtRAD51 paralogs AtRAD51C and AtXRCC3. Most AtRAD51 foci are required for DNA repair using either homologs or sister chromatids as templates without CO formation, while a small number of AtRAD51 foci might play a role in normal CO formation dependent also on AtDMC1. Therefore, the AtRAD51C-AtXRCC3 is critical for ensuring wild type number of AtRAD51 foci and COs and facilitating proper homolog recombination and association.

### A model for the role of RAD51 paralogs in meiotic recombination

**Fig 9:**
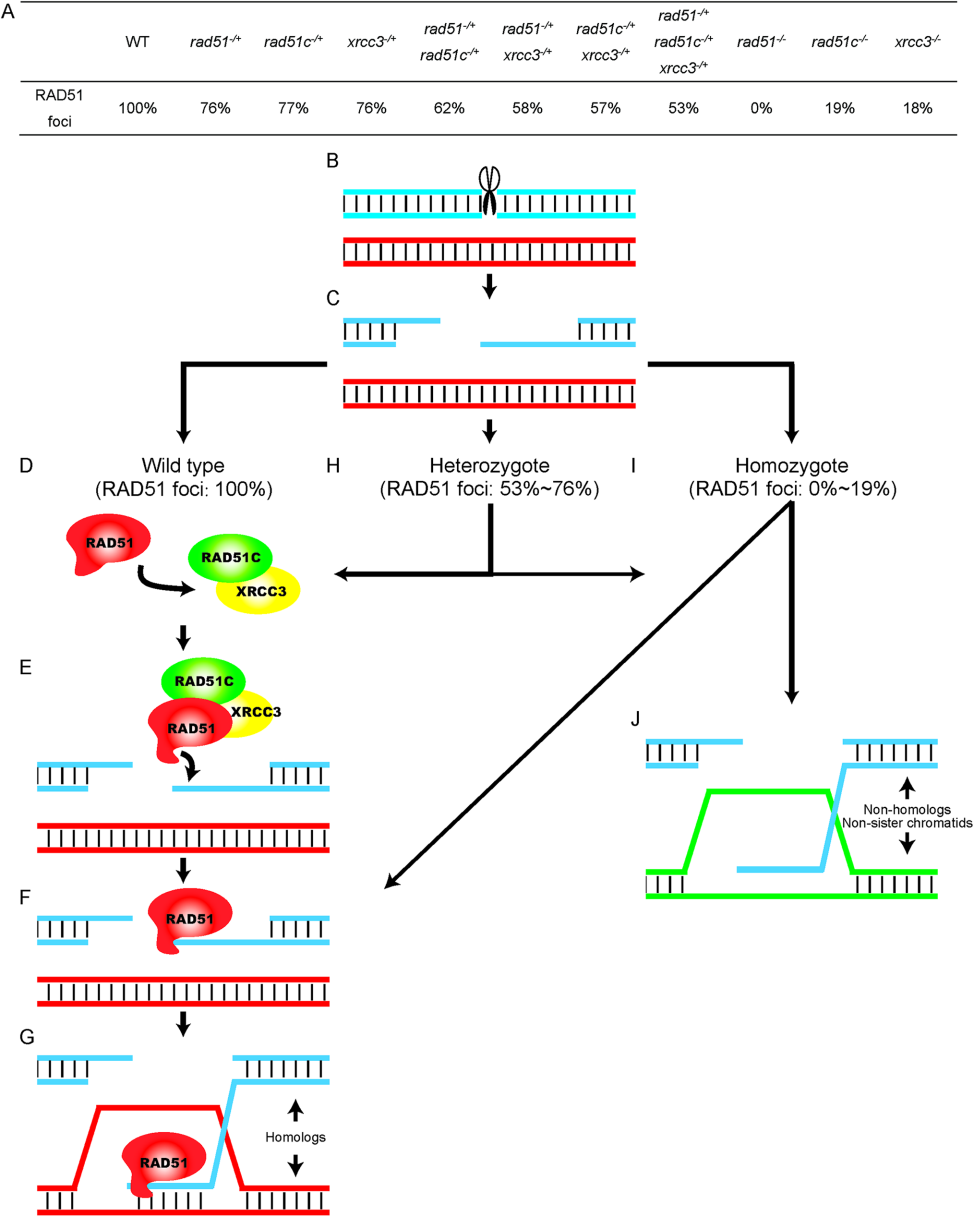
Model of the role of RAD51C- and XRCC3-mediated meiotic recombination (A) The number of RAD51 foci observed in different genotypes at zygotene. (B) Meiotic recombination is initiated by the formation of double-strand breaks (DSBs), catalyzed by SPO11. (C) DSB ends are processed by the MRE11- RAD50-NBS1 (MRN) protein complexes to generate 3′ ssDNA tails [2,3]. (D) In wild type, with normal RAD51 function, interactions between the RAD51C-XRCC3 complex and RAD51 may alter the configuration of RAD51 proteins and facilitate its binding to the single-strand DNA tails (E), and formation of nucleoprotein filaments (F). (G) The RAD51-mediated single end invasion results in formation of a recombination intermediate called a D-loop. (H) In the heterozygous mutant backgrounds, insufficient levels of RAD51C and XRCC3 lead to reduced RAD51 binding, leading to homolog invasion when RAD51 binds (G) or non-homolog invasion (J) in the absence of RAD51. (I-J) In the homozygous mutants, due to the absence, or severe reduction of RAD51, the single-ended DNA may form joint molecules with homologs, non-homologs or sister chromatids. The weight of the arrows indicates the proportional balance of the various intermediate products.

Based on our results and previous studies, we propose a model for how AtRAD51C and AtXRCC3 function in conjunction with AtRAD51 in meiotic HR (Fig 9). Meiotic recombination is initiated by programmed DSBs that are catalyzed by AtSPO11-1 and other proteins. The broken ends are further processed by the MRX protein complex to produce ssDNA tails [2,75–77]. In WT, interaction between the AtRAD51C-AtXRCC3 complex and AtRAD51 is proposed to alter the latter’s configuration and facilitates its binding with the ssDNA tails, thus resulting in single end invasion. Consequently, repair of the DSBs yields either COs or NCOs. In the heterozygous mutants, the reduced AtRAD51 level is likely insufficient for supporting the AtDMC1 function, consistent with previous studies in both *Arabidopsis* and yeast showing that normal *DMC1* function in meiosis requires *RAD51* [6,78]. Thus, with reduced amounts of RAD51 proteins, single end invasion is possibly more promiscuous and targets both homologous and non-homologous templates, resulting in multivalent formation. This aspect of the model is supported by the observation that the triple heterozygous mutant and the weak *atrad51* mutant had non-homologous associations and reduced COs. In the homozygous mutants, when AtRAD51 is either completely absent or reduced below a threshold, most or all DSBs are unrepaired, leading to severe chromosome fragmentation and chromosome entanglements. Further investigations are needed to establish the precise AtRAD51 thresholds and how the AtRAD51 paralogs maintain the necessary level of AtRAD51 during the single-end invasion process.

In summary, meiotic DSB repair is essential for sexual reproduction in eukaryotes including budding yeast, animals and flowering plants. RAD51 paralogs facilitate the establishment of RAD51 at DSBs and mediate and single end invasion. These functions are also highly conserved in eukaryotes. We propose that facilitation of normal RAD51 function by its paralogs, such as RAD51C and XRCC3, may be a general mechanism for meiotic DSB repair.

## Materials and methods

### Plant material and genotyping

The mutants *atrad51-3* (SAIL_873_C08) [42], *atrad51c* (SALK_021960) [13], *atxrcc3* (SALK_045564) [14] used in this study were shown previously to be null mutants in the Columbia (Col-0) background. *atrad51*^*-/+*^ *atrad51c*^*-/+*^ and *atrad51*^*-/-*^ *atrad51c*^*-/-*^ mutants were crossed by *atrad51*^*-/+*^ (male parent) and *atrad51c*^*-/+*^ (female parent), *atrad51*^*-/+*^ *xrcc3*^*-/+*^ and *atrad51*^*-/-*^ *xrcc3*^*-/-*^ mutants were crossed by *atrad51*^*-/+*^ (male parent) and *xrcc3*^*-/+*^ (female parent), *atrad51c*^*-/+*^ *xrcc3*^*-/+*^ and *atrad51c*^*-/-*^ *xrcc3*^*-/-*^ mutants were crossed by *atrad51c*^*-/+*^ (male parent) and *xrcc3*^*-/+*^ (female parent). Triple heterozygous mutants were crossed by *atrad51c*^*-/+*^ (male parent) and *atrad51*^*-/+*^ *atxrcc3*^*-/+*^ (female parent). Plants were grown at 21°C with 16 h light and 8 h dark. Mutant genotypes were confirmed by PCR using the primers described in S2 Table.

### Phenotype analysis

A minimum of 10 plants were characterized for each mutant. Chromosome spreads were stained with DAPI and centromere FISH, and immuno-localization experiments were carried out as described previously [79]. Rabbit polyclonal AtRAD51 and γ-H2AX antibodies were used at 1:200 fold dilutions and Alexa Fluor 488 Goat Anti-Rat IgG (H+L) secondary antibody (A-21428, Invitrogen, Carlsbad, CA, USA) was used at a 1:1000 fold dilution [80]. Chiasmata distribution statistics were performed following the protocol of Sanchez *et al*. [81]. BAC DNA extraction (F19K16) and probe labeling were described previously [43]. Images of chromosome spreads were obtained using an Axio Imager A2 microscope (Zeiss, Heidelberg, Germany) equipped with a digital camera (Canon, Tokyo, Japan), and processed using Photoshop CS (Adobe Systems, Mountain View, CA). Images were initially captured in black & white and, if necessary, globally false-colored post-capture for visual contrast. AtRAD51 and γ-H2AX foci in WT and mutant lines were counted and statistically analyzed using ImageTool version 3.0 software (University of Texas Health Science Center, San Antonio, USA).

In mutants that lacked synapsis, we distinguished zygotene from pachytene chromosomes by their relative condensation, with pachytene being more condensed than zygotene chromosomes.

### Constructs

To construct the vectors for yeast two-hybrid, pull-down and BiFC assays, full-length *AtRAD51*, *AtRAD51C* and *AtXRCC3* cDNA were PCR-amplified using Phanta Super-Fidelity DNA polymerase (Vazyme Biotech Co., Ltd, China) and appropriate primers (S2 Table).

For the Y2H assay, full-length *AtRAD51* and *AtXRCC3* cDNA were purified and ligated into pGADT7 pGBKT7 by *Nde*I and *Bam*HI double-enzyme digestion, and full-length *AtRAD51C* cDNA was purified and ligated into pGADT7 and pGBKT7 by *Nde*I and *Eco*RI double-enzyme digestion.

For the BiFC assay, full-length *AtRAD51* and *AtXRCC3* cDNA was purified and ligated into pXY103, pXY104, pXY105 and pXY106 by *Bam*HI and *Sal*I double-enzyme digestion, and full-length *AtRAD51C* cDNA was purified and ligated into pXY103, pXY104, pXY105 and pXY106 by *Xba*I and *Sal*I double-enzyme digestion.

For the pull down assay, full-length *AtRAD51* and *AtXRCC3* cDNA was purified and ligated into pET32a and pGEX-6P-1 by *Bam*HI and *Sal*I double-enzyme digestion and full-length *AtRAD51C* cDNA was purified and ligated into pET32a and pGEX-6P-1 by *Eco*RI and *Sal*I double-enzyme digestion. All constructs were verified by DNA sequencing.

### Yeast two-hybrid assay

Plasmid vectors were transformed into the Y2H gold yeast strain (pGBKT7 constructs) or the Y187 yeast strain (pGADT7 constructs) using the LiAc/PEG method. Transformants were mated on YPDA medium for 48 h, and selected on SD/–Trp–Leu plates for 36 h. Transformants were then selected on SD/–His–Ade–Trp–Leu with X-α-Gal and AbA plates to test for positive interactions [82].

### Pull-down assay

AtRAD51, AtRAD51C and AtXRCC3 were expressed in *E*. *coli* using the pGEX6P-1 and pET32a plasmids. The tagged proteins were mixed and incubated for 2 h at 4°C, then pulled down by GST beads for 1 h at 4°C. The protein mixture was confirmed by western blotting with a GST antibody (AG768, Beyotime Co. Ltd, China) or a His-tag antibody (AH367, Beyotime Co. Ltd, China) at 1:100 dilutions, followed by application of an horseradish peroxidase (HRP) goat anti-mouse IgG (H+L) secondary antibody (A0216, Beyotime Co. Ltd, China) at a 1:2000 dilution.

### BiFC assay

BiFC plasmids (pXY103/104/105/106-RAD51, pXY103/104/105/106-RAD51C, pXY103/104/105/106-XRCC3 and pXY103/104/105/106) were transformed into *Agrobacterium* GV3101 cells. Transformants were harvested once the OD_600_ reached 2.0, and resuspended in MES/MgCl_2_/acetosyringone solution to a final OD_600_ of 1.0. Cell suspensions were mixed in 1:1 ratios of various combinations, and young *Nicotiana benthamiana* leaves were infiltrated. Leaves were excised and visualized using a LSM-710 confocal microscope (Zeiss) following 36 h incubation [83].

### Flow cytometry

Open flowers from WT plants or *atrad51*^*-/+*^ *atrad51c*^*-/+*^ *atxrcc3*^*-/+*^plants that were hemizygous for the fluorescent-tagged line (FTL) interval I2b and either *QRT*^*+/+*^ or *qrt*^*-/+*^ were collected [64]. The flowers (50 or more) were mixed with 1 mL PBS buffer (10 mM CaCl_2_, 1 mM KCl, 2 mM MES, 5% w/v sucrose, pH 6.5) supplemented with 0.01% Triton X-100 in a 1.5-mL microcentrifuge tube. The mixture was vortexed at maximum speed for 2–3 min and the solution filtered through a 70-μm Falcon^®^ cell strainer (352350, Corning Life Sciences, Tewksbury, MA, USA) at 450 ×*g* for 2 min at 4°C. The flow-through was resuspended in a fresh tube with 1 mL PBS buffer at 4°C. Flow cytometry analysis was performed using a Gallios flow cytometer (Beckman Coulter, Inc.). Statistical analysis was performed using Kaluza Analysis 1.3 software (Beckman Coulter, Inc.) using the two-color analysis methods described previously [65,84].

### Statistical methods

Excel 2016 (Microsoft, USA) was used to calculate the mean and standard error of the AtRAD51 foci, γ-H2AX foci, MLH1 foci and the chiasmata numbers of WT and mutants. Data was compared using Student’s *t*-tests and P values were reported as either exact values or Gaussian approximations.

## Supporting information

S1 Fig Chromosome morphologies in *atrad51*^*-/-*^ *atrad51c*^*-/-*^, *atrad51*^*-/-*^ *atxrcc3*^*-/-*^ and *atrad51c*^*-/-*^ *atxrcc3*^*-/-*^ double homozygous mutants. (A-T) Wild-type (WT), *atrad51*^*-/-*^ *atrad51c*^*-/-*^, *atrad51*^*-/-*^ *atxrcc3*^*-/-*^ and *atrad51c*^*-/-*^ *atxrcc3*^*-/-*^ mutant chromosome morphologies at pachytene, diakinesis, anaphase I, anaphase II and tetrad formation. In comparison with single homozygotes, *atrad51*^*-/-*^ *atrad51c*^*-/-*^, *atrad51*^*-/-*^ *atxrcc3*^*-/-*^ and *atrad51c*^*-/-*^ *atxrcc3*^*-/-*^ mutants had similar chromosome phenotypes. Scale bar: 5 μm.
(PDF)

S2 Fig Immunofluorescence of ASY1 at zygotene and ZYP1 at pachytene in wild type, *atrad51*, *atrad51c* and *atxrcc3* mutants. (A) Localization of ASY1 on wild-type (WT) chromosomes at zygotene. (B-D) Immunofluorescence of ASY1 at zygotene in *atrad51*, *atrad51c* and *atxrcc3* mutants. (E) Localization of ZYP1 on wild-type (WT) chromosomes at pachytene. (F-H) Localization of ZYP1 at pachytene in *atrad51*, *atrad51c* and *atxrcc3* mutants. Left panels show the chromosome morphology following staining with 6-diamidino-2-phenylindole (DAPI), middle panels show ASY1 signal (red line) or ZYP1 signal (red point/line), and right panels merge the DAPI-stained images with the ASY1/ZYP1 signal images. Scale bar: 5 μm.
(PDF)

S3 Fig Fluorescence *in situ* hybridization analysis of chromosome behavior in *atrad51*^*-/+*^, *atrad51c*^*-/+*^ and *atxrcc3*^*-/+*^ single heterozygous mutants. (A-P) Wild type (WT), *atrad51*^*-/+*^, *atrad51c*^*-/+*^ and *atxrcc3*^*-/+*^ mutant chromosome morphologies and centromere signals (shown as red dots) at pachytene, diakinesis, metaphase I and tetrad formation. In comparison with WT, *atrad51*^*-/+*^, *atrad51c*^*-/+*^ and *atxrcc3*^*-/+*^ mutants had similar chromosome phenotypes. Scale bar: 5 μm.
(PDF)

S4 Fig Immunostaining of DMC1 signals in single, double and triple heterozygous mutant chromosomes at zygotene and pachytene stages. The distribution of DMC1 among the eight genotypes examined shows no obvious differences on zygotene (A-H) and pachytene (I-P) chromosomes. Left panels show the chromosome morphology following staining with 6-diamidino-2-phenylindole (DAPI), middle panels show DMC1 foci (red dots), and right panels merge the DAPI-stained images with the DMC1 foci images. (Q-R) The number of DMC1 foci in chromosomes from the eight genotypes at zygotene and pachytene in A-P. Scale bar: 5 μm.
(PDF)

S1 Table P-values of numbers of γ-H2AX foci for all comparisons at zygotene and pachytene. (PDF)

S2 Table Primers used in this study. (XLSX)

## References

[1] Keeney S, Giroux CN, Kleckner N (1997) Meiosis-specific DNA double-strand breaks are catalyzed by Spo11, a member of a widely conserved protein family. Cell 88: 375–384. 9039264 10.1016/s0092-8674(00)81876-0

[2] Amiard S, Charbonnel C, Allain E, Depeiges A, White CI, et al (2010) Distinct roles of the ATR kinase and the Mre11-Rad50-Nbs1 complex in the maintenance of chromosomal stability in Arabidopsis. Plant Cell 22: 3020–3033. doi: 10.1105/tpc.110.078527 20876831 10.1105/tpc.110.078527 PMC2965537

[3] Paull TT, Gellert M (1999) Nbs1 potentiates ATP-driven DNA unwinding and endonuclease cleavage by the Mre11/Rad50 complex. Genes Dev 13: 1276–1288. 10346816 10.1101/gad.13.10.1276 PMC316715

[4] Gasior SL, Wong AK, Kora Y, Shinohara A, Bishop DK (1998) Rad52 associates with RPA and functions with rad55 and rad57 to assemble meiotic recombination complexes. Genes Dev 12: 2208–2221. 9679065 10.1101/gad.12.14.2208 PMC317010

[5] Muller B, Koller T, Stasiak A (1990) Characterization of the DNA binding activity of stable RecA-DNA complexes. Interaction between the two DNA binding sites within RecA helical filaments. J Mol Biol 212: 97–112. doi: 10.1016/0022-2836(90)90307-8 2319601 10.1016/0022-2836(90)90307-8

[6] Cloud V, Chan YL, Grubb J, Budke B, Bishop DK (2012) Rad51 is an accessory factor for Dmc1-mediated joint molecule formation during meiosis. Science 337: 1222–1225. doi: 10.1126/science.1219379 22955832 10.1126/science.1219379 PMC4056682

[7] Sung P (1997) Function of yeast Rad52 protein as a mediator between replication protein A and the Rad51 recombinase. J Biol Chem 272: 28194–28197. 9353267 10.1074/jbc.272.45.28194

[8] Petukhova G, Stratton S, Sung P (1998) Catalysis of homologous DNA pairing by yeast Rad51 and Rad54 proteins. Nature 393: 91–94. doi: 10.1038/30037 9590697 10.1038/30037

[9] Shinohara M, Gasior SL, Bishop DK, Shinohara A (2000) Tid1/Rdh54 promotes colocalization of rad51 and dmc1 during meiotic recombination. Proc Natl Acad Sci U S A 97: 10814–10819. 11005857 10.1073/pnas.97.20.10814 PMC27106

[10] Sung P (1997) Yeast Rad55 and Rad57 proteins form a heterodimer that functions with replication protein A to promote DNA strand exchange by Rad51 recombinase. Genes Dev 11: 1111–1121. 9159392 10.1101/gad.11.9.1111

[11] Haruta N, Kurokawa Y, Murayama Y, Akamatsu Y, Unzai S, et al (2006) The Swi5-Sfr1 complex stimulates Rhp51/Rad51- and Dmc1-mediated DNA strand exchange in vitro. Nat Struct Mol Biol 13: 823–830. doi: 10.1038/nsmb1136 16921379 10.1038/nsmb1136

[12] Sasanuma H, Tawaramoto MS, Lao JP, Hosaka H, Sanda E, et al (2013) A new protein complex promoting the assembly of Rad51 filaments. Nat Commun 4: 1676 doi: 10.1038/ncomms2678 23575680 10.1038/ncomms2678 PMC4353811

[13] Li W, Yang X, Lin Z, Timofejeva L, Xiao R, et al (2005) The AtRAD51C gene is required for normal meiotic chromosome synapsis and double-stranded break repair in Arabidopsis. Plant Physiol 138: 965–976. doi: 10.1104/pp.104.058347 15923332 10.1104/pp.104.058347 PMC1150411

[14] Bleuyard JY, White CI (2004) The Arabidopsis homologue of Xrcc3 plays an essential role in meiosis. EMBO J 23: 439–449. doi: 10.1038/sj.emboj.7600055 14726957 10.1038/sj.emboj.7600055 PMC1271761

[15] Vignard J, Siwiec T, Chelysheva L, Vrielynck N, Gonord F, et al (2007) The interplay of RecA-related proteins and the MND1-HOP2 complex during meiosis in Arabidopsis thaliana. PLoS Genet 3: 1894–1906. doi: 10.1371/journal.pgen.0030176 17937504 10.1371/journal.pgen.0030176 PMC2014788

[16] Kurzbauer M-T, Uanschou C, Chen D, Schlögelhofer P (2012) The recombinases DMC1 and RAD51 are functionally and spatially separated during meiosis in Arabidopsis. The Plant Cell 24: 2058–2070. doi: 10.1105/tpc.112.098459 22589466 10.1105/tpc.112.098459 PMC3442587

[17] Petukhova GV, Pezza RJ, Vanevski F, Ploquin M, Masson JY, et al (2005) The Hop2 and Mnd1 proteins act in concert with Rad51 and Dmc1 in meiotic recombination. Nat Struct Mol Biol 12: 449–453. doi: 10.1038/nsmb923 15834424 10.1038/nsmb923

[18] Yang H, Jeffrey PD, Miller J, Kinnucan E, Sun Y, et al (2002) BRCA2 function in DNA binding and recombination from a BRCA2-DSS1-ssDNA structure. Science 297: 1837–1848. doi: 10.1126/science.297.5588.1837 12228710 10.1126/science.297.5588.1837

[19] Hunter N, Kleckner N (2001) The single-end invasion: an asymmetric intermediate at the double-strand break to double-holliday junction transition of meiotic recombination. Cell 106: 59–70. 11461702 10.1016/s0092-8674(01)00430-5

[20] Lin Z, Kong H, Nei M, Ma H (2006) Origins and evolution of the recA/RAD51 gene family: evidence for ancient gene duplication and endosymbiotic gene transfer. Proc Natl Acad Sci U S A 103: 10328–10333. doi: 10.1073/pnas.0604232103 16798872 10.1073/pnas.0604232103 PMC1502457

[21] Suwaki N, Klare K, Tarsounas M (2011) RAD51 paralogs: roles in DNA damage signalling, recombinational repair and tumorigenesis. Semin Cell Dev Biol 22: 898–905. doi: 10.1016/j.semcdb.2011.07.019 21821141 10.1016/j.semcdb.2011.07.019

[22] Bleuyard JY, Gallego ME, White CI (2006) Recent advances in understanding of the DNA double-strand break repair machinery of plants. DNA Repair (Amst) 5: 1–12. 16202663 10.1016/j.dnarep.2005.08.017

[23] Karpenshif Y, Bernstein KA (2012) From yeast to mammals: recent advances in genetic control of homologous recombination. DNA Repair (Amst) 11: 781–788. 22889934 10.1016/j.dnarep.2012.07.001 PMC3468695

[24] Bleuyard JY, Gallego ME, Savigny F, White CI (2005) Differing requirements for the Arabidopsis Rad51 paralogs in meiosis and DNA repair. Plant J 41: 533–545. doi: 10.1111/j.1365-313X.2004.02318.x 15686518 10.1111/j.1365-313X.2004.02318.x

[25] Pittman DL, Schimenti JC (2000) Midgestation lethality in mice deficient for the RecA-related gene, Rad51d/Rad51l3. Genesis 26: 167–173. 10705376 10.1002/(sici)1526-968x(200003)26:3<167::aid-gene1>3.0.co;2-m

[26] Kuznetsov SG, Haines DC, Martin BK, Sharan SK (2009) Loss of Rad51c leads to embryonic lethality and modulation of Trp53-dependent tumorigenesis in mice. Cancer Res 69: 863–872. doi: 10.1158/0008-5472.CAN-08-3057 19155299 10.1158/0008-5472.CAN-08-3057 PMC2754281

[27] Shu Z, Smith S, Wang L, Rice MC, Kmiec EB (1999) Disruption of muREC2/RAD51L1 in mice results in early embryonic lethality which can Be partially rescued in a p53(-/-) background. Mol Cell Biol 19: 8686–8693. 10567591 10.1128/mcb.19.12.8686 PMC85012

[28] Lim DS, Hasty P (1996) A mutation in mouse rad51 results in an early embryonic lethal that is suppressed by a mutation in p53. Mol Cell Biol 16: 7133–7143. 8943369 10.1128/mcb.16.12.7133 PMC231717

[29] Deans B, Griffin CS, Maconochie M, Thacker J (2000) Xrcc2 is required for genetic stability, embryonic neurogenesis and viability in mice. EMBO J 19: 6675–6685. doi: 10.1093/emboj/19.24.6675 11118202 10.1093/emboj/19.24.6675 PMC305908

[30] Couteau Florence, Belzile Francis, Horlow Christine, Grandjean Olivier, Vezon Daniel, et al (1999) Random Chromosome Segregation without Meiotic Arrest in Both Male and Female eiocytes of a dmc1 Mutant of Arabidopsis. The Plant Cell 11: 1623–1634. 10488231 10.1105/tpc.11.9.1623 PMC144309

[31] Li W, Chen C, Markmann-Mulisch U, Timofejeva L, Schmelzer E, et al (2004) The Arabidopsis AtRAD51 gene is dispensable for vegetative development but required for meiosis. Proc Natl Acad Sci U S A 101: 10596–10601. doi: 10.1073/pnas.0404110101 15249667 10.1073/pnas.0404110101 PMC489980

[32] Abe K, Osakabe K, Nakayama S, Endo M, Tagiri A, et al (2005) Arabidopsis RAD51C gene is important for homologous recombination in meiosis and mitosis. Plant Physiol 139: 896–908. doi: 10.1104/pp.105.065243 16169964 10.1104/pp.105.065243 PMC1256004

[33] Wang Y, Xiao R, Wang H, Cheng Z, Li W, et al (2014) The Arabidopsis RAD51 paralogs RAD51B, RAD51D and XRCC2 play partially redundant roles in somatic DNA repair and gene regulation. New Phytol 201: 292–304. doi: 10.1111/nph.12498 24102485 10.1111/nph.12498

[34] Klimyuk VI, Jones JD (1997) AtDMC1, the Arabidopsis homologue of the yeast DMC1 gene: characterization, transposon-induced allelic variation and meiosis-associated expression. Plant J 11: 1–14. 9025299 10.1046/j.1365-313x.1997.11010001.x

[35] Couteau F, Belzile F, Horlow C, Grandjean O, Vezon D, et al (1999) Random chromosome segregation without meiotic arrest in both male and female meiocytes of a dmc1 mutant of Arabidopsis. Plant Cell 11: 1623–1634. 10488231 10.1105/tpc.11.9.1623 PMC144309

[36] Wiese C, Collins DW, Albala JS, Thompson LH, Kronenberg A, et al (2002) Interactions involving the Rad51 paralogs Rad51C and XRCC3 in human cells. Nucleic Acids Res 30: 1001–1008. 11842112 10.1093/nar/30.4.1001 PMC100332

[37] Masson JY, Stasiak AZ, Stasiak A, Benson FE, West SC (2001) Complex formation by the human RAD51C and XRCC3 recombination repair proteins. Proc Natl Acad Sci U S A 98: 8440–8446. doi: 10.1073/pnas.111005698 11459987 10.1073/pnas.111005698 PMC37455

[38] Bishop DK, Ear U, Bhattacharyya A, Calderone C, Beckett M, et al (1998) Xrcc3 is required for assembly of Rad51 complexes in vivo. Journal of Biological Chemistry 273: 21482–21488. 9705276 10.1074/jbc.273.34.21482

[39] Liu N, Schild D, Thelen MP, Thompson LH (2002) Involvement of Rad51C in two distinct protein complexes of Rad51 paralogs in human cells. Nucleic Acids Res 30: 1009–1015. 11842113 10.1093/nar/30.4.1009 PMC100342

[40] Liu Y, Masson JY, Shah R, O'Regan P, West SC (2004) RAD51C is required for Holliday junction processing in mammalian cells. Science 303: 243–246. doi: 10.1126/science.1093037 14716019 10.1126/science.1093037

[41] Osakabe K, Yoshioka T, Ichikawa H, Toki S (2002) Molecular cloning and characterization of RAD51-like genes from Arabidopsis thaliana. Plant Mol Biol 50: 71–81. 12139010 10.1023/a:1016047231597

[42] Pradillo M, López E, Linacero R, Romero C, Cuñado N, et al (2012) Together yes, but not coupled: new insights into the roles of RAD51 and DMC1 in plant meiotic recombination. The Plant Journal 69: 921–933. doi: 10.1111/j.1365-313X.2011.04845.x 22066484 10.1111/j.1365-313X.2011.04845.x

[43] Wang Y, Cheng Z, Huang J, Shi Q, Hong Y, et al (2012) The DNA replication factor RFC1 is required for interference-sensitive meiotic crossovers in Arabidopsis thaliana. PLoS Genet 8: e1003039 doi: 10.1371/journal.pgen.1003039 23144629 10.1371/journal.pgen.1003039 PMC3493451

[44] Da Ines O, Abe K, Goubely C, Gallego ME, White CI (2012) Differing requirements for RAD51 and DMC1 in meiotic pairing of centromeres and chromosome arms in Arabidopsis thaliana. PLoS Genet 8: e1002636 doi: 10.1371/journal.pgen.1002636 22532804 10.1371/journal.pgen.1002636 PMC3330102

[45] Shinohara A, Ogawa T (1998) Stimulation by Rad52 of yeast Rad51-mediated recombination. Nature 391: 404–407. doi: 10.1038/34943 9450759 10.1038/34943

[46] Liu J, Renault L, Veaute X, Fabre F, Stahlberg H, et al (2011) Rad51 paralogues Rad55-Rad57 balance the antirecombinase Srs2 in Rad51 filament formation. Nature 479: 245–248. doi: 10.1038/nature10522 22020281 10.1038/nature10522 PMC3213327

[47] Jensen RB, Carreira A, Kowalczykowski SC (2010) Purified human BRCA2 stimulates RAD51-mediated recombination. Nature 467: 678–683. doi: 10.1038/nature09399 20729832 10.1038/nature09399 PMC2952063

[48] Seeliger K, Dukowic-Schulze S, Wurz-Wildersinn R, Pacher M, Puchta H (2012) BRCA2 is a mediator of RAD51- and DMC1-facilitated homologous recombination in Arabidopsis thaliana. New Phytol 193: 364–375. doi: 10.1111/j.1469-8137.2011.03947.x 22077663 10.1111/j.1469-8137.2011.03947.x

[49] Li X, Qian W, Zhao Y, Wang C, Shen J, et al (2012) Antisilencing role of the RNA-directed DNA methylation pathway and a histone acetyltransferase in *Arabidopsis*. Proc Natl Acad Sci U S A 109: 11425–11430. doi: 10.1073/pnas.1208557109 22733760 10.1073/pnas.1208557109 PMC3396497

[50] Mercier R, Mezard C, Jenczewski E, Macaisne N, Grelon M (2015) The molecular biology of meiosis in plants. Annu Rev Plant Biol 66: 297–327. doi: 10.1146/annurev-arplant-050213-035923 25494464 10.1146/annurev-arplant-050213-035923

[51] Lowndes NF, Toh GW (2005) DNA repair: the importance of phosphorylating histone H2AX. Curr Biol 15: R99–R102. doi: 10.1016/j.cub.2005.01.029 15694301 10.1016/j.cub.2005.01.029

[52] Masson JY, Tarsounas MC, Stasiak AZ, Stasiak A, Shah R, et al (2001) Identification and purification of two distinct complexes containing the five RAD51 paralogs. Genes Dev 15: 3296–3307. doi: 10.1101/gad.947001 11751635 10.1101/gad.947001 PMC312846

[53] Huffaker TC, Hoyt MA, Botstein D (1987) Genetic analysis of the yeast cytoskeleton. Annual review of genetics 21: 259–284. doi: 10.1146/annurev.ge.21.120187.001355 3327466 10.1146/annurev.ge.21.120187.001355

[54] Phizicky EM, Fields S (1995) Protein-protein interactions: methods for detection and analysis. Microbiol Rev 59: 94–123. 7708014 10.1128/mr.59.1.94-123.1995 PMC239356

[55] Crismani W, Mercier R (2013) Identifying meiotic mutants in Arabidopsis thaliana. Plant Meiosis: Methods and Protocols: 227–234. 10.1007/978-1-62703-333-6_22 23559218

[56] Clark KA, Krysan PJ (2010) Chromosomal translocations are a common phenomenon in Arabidopsis thaliana T‐DNA insertion lines. The Plant Journal 64: 990–1001. doi: 10.1111/j.1365-313X.2010.04386.x 21143679 10.1111/j.1365-313X.2010.04386.x PMC3079379

[57] Higgins JD, Armstrong SJ, Franklin FC, Jones GH (2004) The Arabidopsis MutS homolog AtMSH4 functions at an early step in recombination: evidence for two classes of recombination in Arabidopsis. Genes Dev 18: 2557–2570. doi: 10.1101/gad.317504 15489296 10.1101/gad.317504 PMC529542

[58] Jackson N, Sanchez-Moran E, Buckling E, Armstrong SJ, Jones GH, et al (2006) Reduced meiotic crossovers and delayed prophase I progression in AtMLH3-deficient Arabidopsis. EMBO J 25: 1315–1323. doi: 10.1038/sj.emboj.7600992 16467846 10.1038/sj.emboj.7600992 PMC1422170

[59] Chelysheva L, Grandont L, Vrielynck N, le Guin S, Mercier R, et al (2010) An easy protocol for studying chromatin and recombination protein dynamics during Arabidopsis thaliana meiosis: immunodetection of cohesins, histones and MLH1. Cytogenet Genome Res 129: 143–153. doi: 10.1159/000314096 20628250 10.1159/000314096

[60] Berchowitz LE, Copenhaver GP (2010) Genetic interference: don't stand so close to me. Curr Genomics 11: 91–102. doi: 10.2174/138920210790886835 20885817 10.2174/138920210790886835 PMC2874225

[61] Berchowitz LE, Francis KE, Bey AL, Copenhaver GP (2007) The role of AtMUS81 in interference-insensitive crossovers in A. thaliana. PLoS Genet 3: e132 doi: 10.1371/journal.pgen.0030132 17696612 10.1371/journal.pgen.0030132 PMC1941751

[62] Jones GH, Franklin FC (2006) Meiotic crossing-over: obligation and interference. Cell 126: 246–248. doi: 10.1016/j.cell.2006.07.010 16873056 10.1016/j.cell.2006.07.010

[63] Francis KE, Lam SY, Harrison BD, Bey AL, Berchowitz LE, et al (2007) Pollen tetrad-based visual assay for meiotic recombination in Arabidopsis. Proc Natl Acad Sci U S A 104: 3913–3918. doi: 10.1073/pnas.0608936104 17360452 10.1073/pnas.0608936104 PMC1805420

[64] Berchowitz LE, Copenhaver GP (2008) Fluorescent Arabidopsis tetrads: a visual assay for quickly developing large crossover and crossover interference data sets. Nat Protoc 3: 41–50. doi: 10.1038/nprot.2007.491 18193020 10.1038/nprot.2007.491

[65] Yelina NE, Ziolkowski PA, Miller N, Zhao X, Kelly KA, et al (2013) High-throughput analysis of meiotic crossover frequency and interference via flow cytometry of fluorescent pollen in Arabidopsis thaliana. Nat Protoc 8: 2119–2134. doi: 10.1038/nprot.2013.131 24113785 10.1038/nprot.2013.131

[66] Taylor MR, Spirek M, Chaurasiya KR, Ward JD, Carzaniga R, et al (2015) Rad51 Paralogs Remodel Pre-synaptic Rad51 Filaments to Stimulate Homologous Recombination. Cell 162: 271–286. doi: 10.1016/j.cell.2015.06.015 26186187 10.1016/j.cell.2015.06.015 PMC4518479

[67] Liu N, Lamerdin JE, Tebbs RS, Schild D, Tucker JD, et al (1998) XRCC2 and XRCC3, new human Rad51-family members, promote chromosome stability and protect against DNA cross-links and other damages. Mol Cell 1: 783–793. 9660962 10.1016/s1097-2765(00)80078-7

[68] Pierce AJ, Johnson RD, Thompson LH, Jasin M (1999) XRCC3 promotes homology-directed repair of DNA damage in mammalian cells. Genes Dev 13: 2633–2638. 10541549 10.1101/gad.13.20.2633 PMC317094

[69] Lio YC, Schild D, Brenneman MA, Redpath JL, Chen DJ (2004) Human Rad51C deficiency destabilizes XRCC3, impairs recombination, and radiosensitizes S/G2-phase cells. J Biol Chem 279: 42313–42320. doi: 10.1074/jbc.M405212200 15292210 10.1074/jbc.M405212200

[70] Zhang B, Wang M, Tang D, Li Y, Xu M, et al (2015) XRCC3 is essential for proper double-strand break repair and homologous recombination in rice meiosis. J Exp Bot 66: 5713–5725. doi: 10.1093/jxb/erv253 26034131 10.1093/jxb/erv253

[71] Tang D, Miao C, Li Y, Wang H, Liu X, et al (2014) OsRAD51C is essential for double-strand break repair in rice meiosis. Front Plant Sci 5: 167 doi: 10.3389/fpls.2014.00167 24847337 10.3389/fpls.2014.00167 PMC4019848

[72] Wang H, Hu Q, Tang D, Liu X, Du G, et al (2016) OsDMC1 Is Not Required for Homologous Pairing in Rice Meiosis. Plant Physiol 171: 230–241. doi: 10.1104/pp.16.00167 26960731 10.1104/pp.16.00167 PMC4854709

[73] Liu Y, Tarsounas M, O'Regan P, West SC (2007) Role of RAD51C and XRCC3 in genetic recombination and DNA repair. J Biol Chem 282: 1973–1979. doi: 10.1074/jbc.M609066200 17114795 10.1074/jbc.M609066200

[74] Da Ines O, Degroote F, Amiard S, Goubely C, Gallego ME, et al (2013) Effects of XRCC2 and RAD51B mutations on somatic and meiotic recombination in Arabidopsis thaliana. Plant J 74: 959–970. doi: 10.1111/tpj.12182 23521529 10.1111/tpj.12182

[75] grelon M, Vezon D, Gendort G, Pelleiter G (2001) AtSPO11-1 is necessary for eficient meiotic recombination in plant. The EMBO journal 20: 589–600. doi: 10.1093/emboj/20.3.589 11157765 10.1093/emboj/20.3.589 PMC133473

[76] Hamant O, Ma H, Cande WZ (2006) Genetics of meiotic prophase I in plants. Annu Rev Plant Biol 57: 267–302. doi: 10.1146/annurev.arplant.57.032905.105255 16669763 10.1146/annurev.arplant.57.032905.105255

[77] Stacey NJ, Kuromori T, Azumi Y, Roberts G, Breuer C, et al (2006) Arabidopsis SPO11-2 functions with SPO11-1 in meiotic recombination. Plant J 48: 206–216. doi: 10.1111/j.1365-313X.2006.02867.x 17018031 10.1111/j.1365-313X.2006.02867.x

[78] Da Ines O, Degroote F, Goubely C, Amiard S, Gallego ME, et al (2013) Meiotic recombination in Arabidopsis is catalysed by DMC1, with RAD51 playing a supporting role. PLoS Genet 9: e1003787 doi: 10.1371/journal.pgen.1003787 24086145 10.1371/journal.pgen.1003787 PMC3784562

[79] Wang Y, Cheng Z, Lu P, Timofejeva L, Ma H (2014) Molecular cell biology of male meiotic chromosomes and isolation of male meiocytes in Arabidopsis thaliana. Methods Mol Biol 1110: 217–230. doi: 10.1007/978-1-4614-9408-9_10 24395259 10.1007/978-1-4614-9408-9_10

[80] Huang J, Cheng Z, Wang C, Hong Y, Su H, et al (2015) Formation of interference-sensitive meiotic cross-overs requires sufficient DNA leading-strand elongation. Proc Natl Acad Sci U S A 112: 12534–12539. doi: 10.1073/pnas.1507165112 26392549 10.1073/pnas.1507165112 PMC4603498

[81] Sanchez Moran E, Armstrong SJ, Santos JL, Franklin FC, Jones GH (2001) Chiasma formation in Arabidopsis thaliana accession Wassileskija and in two meiotic mutants. Chromosome Res 9: 121–128. 11321367 10.1023/a:1009278902994

[82] Cui J, You C, Zhu E, Huang Q, Ma H, et al (2016) Feedback Regulation of DYT1 by Interactions with Downstream bHLH Factors Promotes DYT1 Nuclear Localization and Anther Development. Plant Cell 28: 1078–1093. doi: 10.1105/tpc.15.00986 27113773 10.1105/tpc.15.00986 PMC4904671

[83] Zhu E, You C, Wang S, Cui J, Niu B, et al (2015) The DYT1-interacting proteins bHLH010, bHLH089 and bHLH091 are redundantly required for Arabidopsis anther development and transcriptome. Plant J 83: 976–990. doi: 10.1111/tpj.12942 26216374 10.1111/tpj.12942

[84] Yelina NE, Choi K, Chelysheva L, Macaulay M, de Snoo B, et al (2012) Epigenetic remodeling of meiotic crossover frequency in Arabidopsis thaliana DNA methyltransferase mutants. PLoS Genet 8: e1002844 doi: 10.1371/journal.pgen.1002844 22876192 10.1371/journal.pgen.1002844 PMC3410864

