# Supplementary figures and images for: Arabidopsis RAD51, RAD51C and XRCC3 proteins form a complex and facilitate RAD51 localization on chromosomes for meiotic recombination
Source: PLoS Genet. 2017 May 31;13(5):e1006827. doi: 10.1371/journal.pgen.1006827 (PMC5470734; doi:10.1371/journal.pgen.1006827)

S1 Fig.

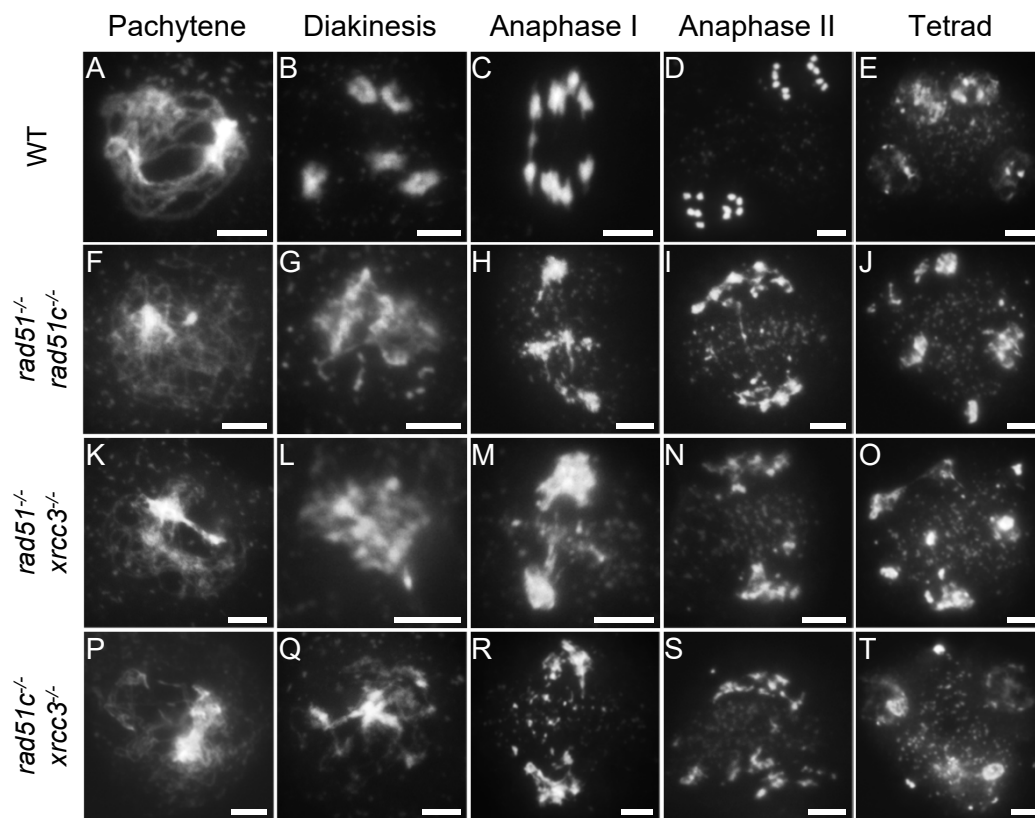

Supplement: S1 Fig — (A-T) Wild-type (WT), atrad51-/- atrad51c-/-, atrad51-/- atxrcc3-/- and atrad51c-/- atxrcc3-/- mutant chromosome morphologies at pachytene, diakinesis, anaphase I, anaphase II and tetrad formation. In comparison with single homozygotes, atrad51-/- atrad51c-/-, atrad51-/- atxrcc3-/- and atrad51c-/- atxrcc3-/- mutants had similar chromosome phenotypes. Scale bar: 5 μm. (PDF) [file pgen.1006827.s001.pdf]

S2 Fig.

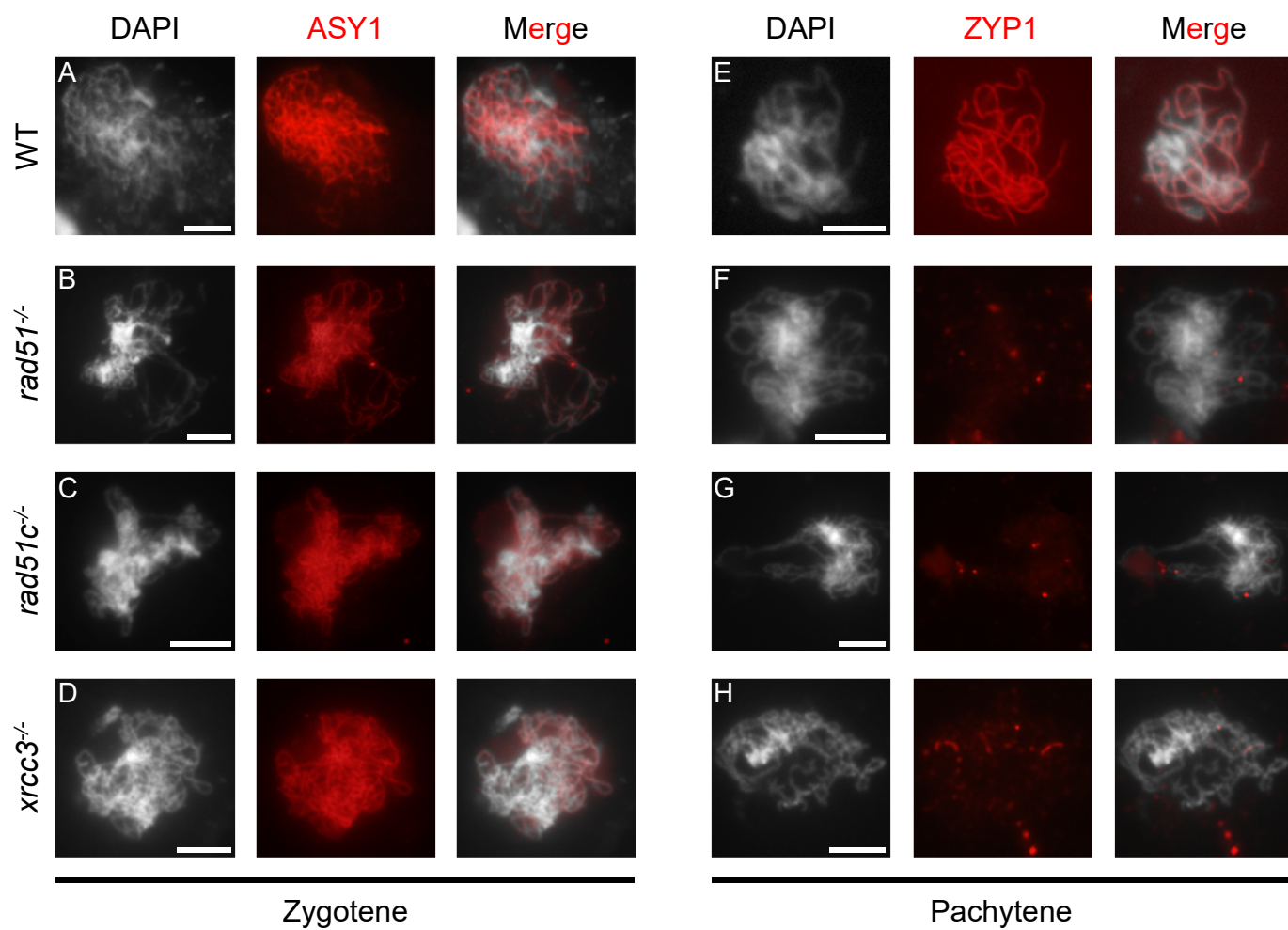

Supplement: S2 Fig — (A) Localization of ASY1 on wild-type (WT) chromosomes at zygotene. (B-D) Immunofluorescence of ASY1 at zygotene in atrad51, atrad51c and atxrcc3 mutants. (E) Localization of ZYP1 on wild-type (WT) chromosomes at pachytene. (F-H) Localization of ZYP1 at pachytene in atrad51, atrad51c and atxrcc3 mutants. Left panels show the chromosome morphology following staining with 6-diamidino-2-phenylindole (DAPI), middle panels show ASY1 signal (red line) or ZYP1 signal (red point/line), and right panels merge the DAPI-stained images with the ASY1/ZYP1 signal images. Scale bar: 5 μm. (PDF) [file pgen.1006827.s002.pdf]

S3 Fig.

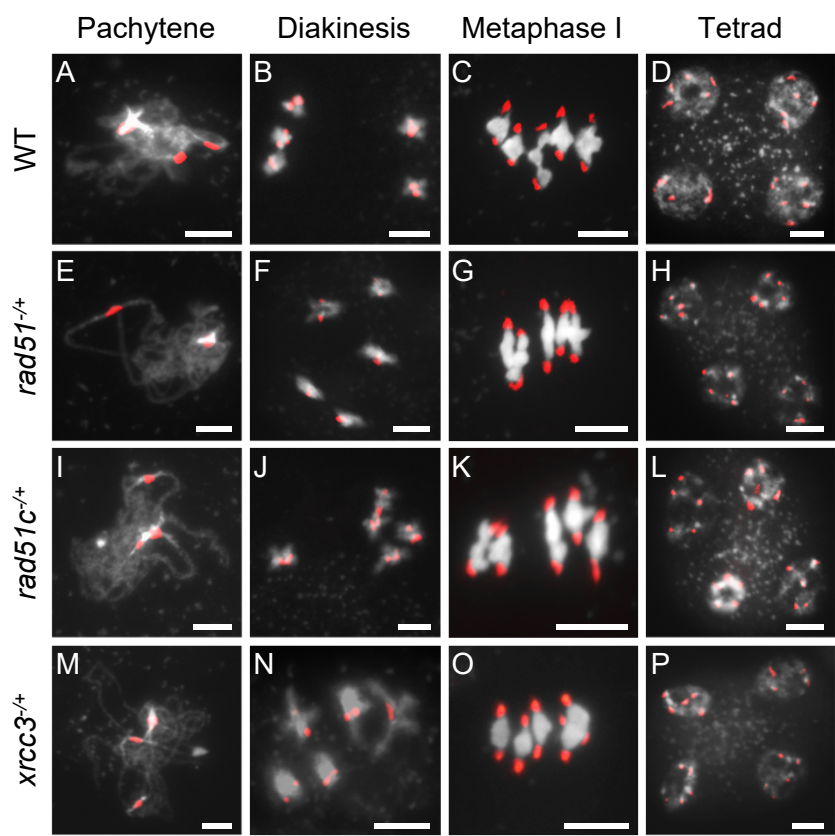

Supplement: S3 Fig — (A-P) Wild type (WT), atrad51-/+, atrad51c-/+ and atxrcc3-/+ mutant chromosome morphologies and centromere signals (shown as red dots) at pachytene, diakinesis, metaphase I and tetrad formation. In comparison with WT, atrad51-/+, atrad51c-/+ and atxrcc3-/+ mutants had similar chromosome phenotypes. Scale bar: 5 μm. (PDF) [file pgen.1006827.s003.pdf]

S4 Fig.

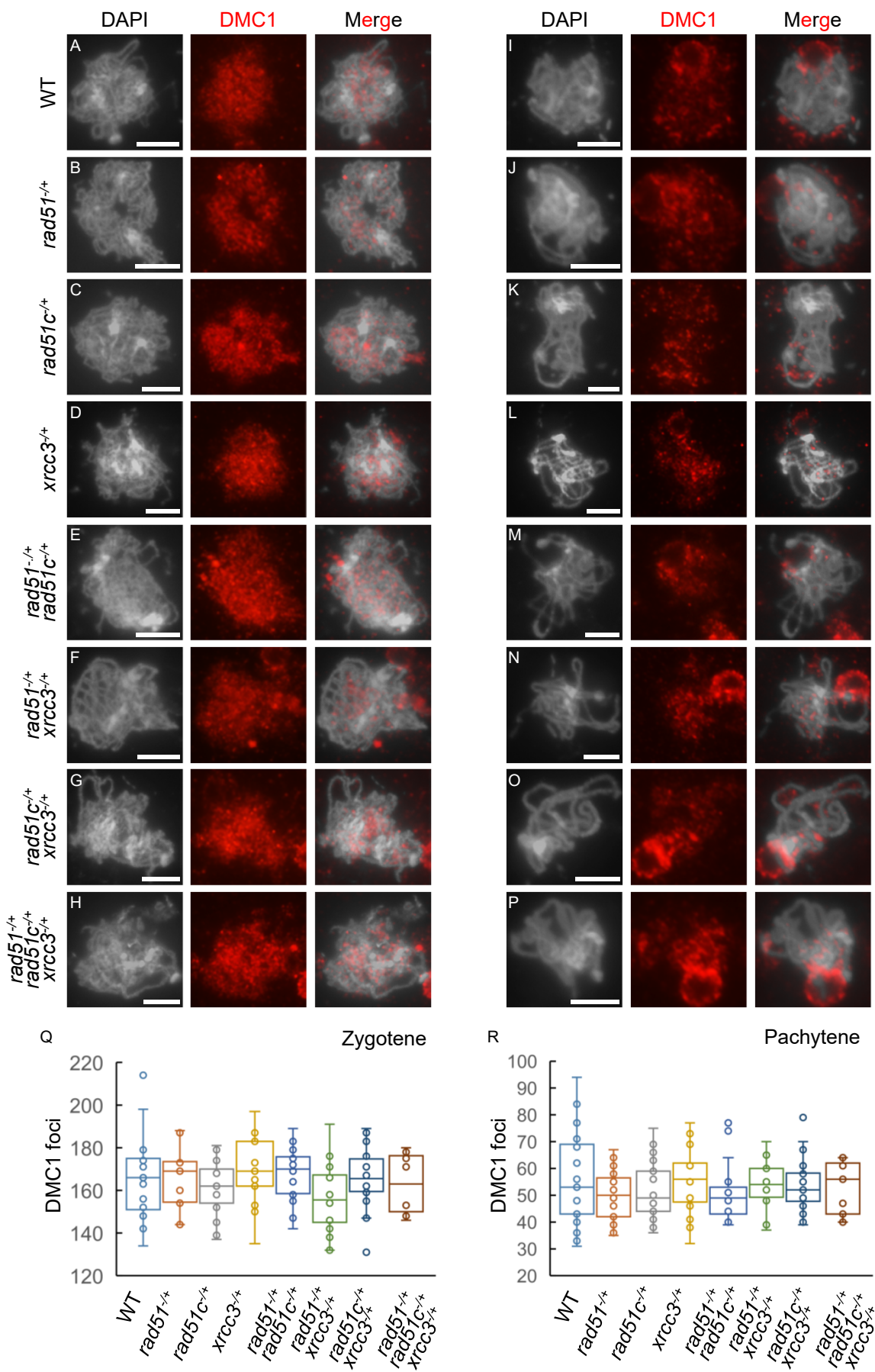

Supplement: S4 Fig — The distribution of DMC1 among the eight genotypes examined shows no obvious differences on zygotene (A-H) and pachytene (I-P) chromosomes. Left panels show the chromosome morphology following staining with 6-diamidino-2-phenylindole (DAPI), middle panels show DMC1 foci (red dots), and right panels merge the DAPI-stained images with the DMC1 foci images. (Q-R) The number of DMC1 foci in chromosomes from the eight genotypes at zygotene and pachytene in A-P. Scale bar: 5 μm. (PDF) [file pgen.1006827.s004.pdf]
