# Supplementary material for: Arabidopsis RAD51, RAD51C and XRCC3 proteins form a complex and facilitate RAD51 localization on chromosomes for meiotic recombination
Source: PLoS Genet. 2017 May 31;13(5):e1006827. doi: 10.1371/journal.pgen.1006827 (PMC5470734; doi:10.1371/journal.pgen.1006827)
Supplement: S1 Table — (PDF) [file pgen.1006827.s005.pdf]

S1 Table

Zygotene	*rad51*$^{-/+}$	*rad51c*$^{-/+}$	*xrcc3*$^{-/+}$	*rad51*$^{-/+}$ *rad51c*$^{-/+}$	*rad51*$^{-/+}$ *xrcc3*$^{-/+}$	*rad51c*$^{-/+}$ *xrcc3*$^{-/+}$	*rad51*$^{-/+}$ *rad51c*$^{-/+}$ *xrcc3*$^{-/+}$
WT	0.100	0.791	0.140	0.0518	0.811	0.633	0.0519
rad51$^{-/+}$		0.242	0.780	0.588	0.123	0.271	0.995
rad51c$^{-/+}$			0.216	0.118	0.659	0.862	0.138
xrcc3$^{-/+}$				0.907	0.145	0.176	0.727
rad51$^{-/+}$ *rad51c*$^{-/+}$					0.0593	0.121	0.496
rad51$^{-/+}$ *xrcc3*$^{-/+}$						0.528	0.0505
rad51c$^{-/+}$ *xrcc3*$^{-/+}$							0.163

Pachytene	*rad51*$^{-/+}$	*rad51c*$^{-/+}$	*xrcc3*$^{-/+}$	*rad51*$^{-/+}$ *rad51c*$^{-/+}$	*rad51*$^{-/+}$ *xrcc3*$^{-/+}$	*rad51c*$^{-/+}$ *xrcc3*$^{-/+}$	*rad51*$^{-/+}$ *rad51c*$^{-/+}$ *xrcc3*$^{-/+}$
WT	0.000426	4.47E-07	0.000120	2.21E-16	1.26E-13	1.05E-06	4.16E-34
rad51$^{-/+}$		0.364	0.587	0.00261	0.000514	0.00177	5.99E-07
rad51c$^{-/+}$			0.829	0.003254	0.000689	0.00507	5.23E-08
xrcc3$^{-/+}$				0.0229	0.00528	0.00785	5.60E-06
rad51$^{-/+}$ *rad51c*$^{-/+}$					0.342	0.206	0.0000114
rad51$^{-/+}$ *xrcc3*$^{-/+}$						0.499	0.00198
rad51c$^{-/+}$ *xrcc3*$^{-/+}$							0.234
